# Simulating lateral distraction osteogenesis

**DOI:** 10.1371/journal.pone.0194500

**Published:** 2018-03-15

**Authors:** Frank Niemeyer, Lutz Claes, Anita Ignatius, Nicholaus Meyers, Ulrich Simon

**Affiliations:** 1 Institute for Orthopaedic Research and Biomechanics, Centre for Trauma Research, University Hospital Ulm, Ulm, Germany; 2 Scientific Computing Centre Ulm (UZWR), Ulm University, Ulm, Germany; Universidad de Zaragoza, SPAIN

## Abstract

Distraction osteogenesis is an effective method for generating large amounts of bone *in situ* for treating pathologies such as large bone defects or skeletal malformations, for instance leg-length discrepancies. While an optimized distraction procedure might have the potential to reduce the rate of complications significantly, our knowledge of the underlying mechanobiological processes is still insufficient for systematic optimization of treatment parameters such as distraction rate or fixation stiffness. We present a novel numerical model of lateral distraction osteogenesis, based on a mechanically well-controlled *in vivo* experiment. This model extends an existing numerical model of callus healing with viscoplastic material properties for describing stress relaxation and stimuli history-dependent tissue differentiation, incorporating delay and memory effects. A reformulation of appositional growth based non-local biological stimuli in terms of spatial convolution as well as remeshing and solution-mapping procedures allow the model to cope with severe mesh distortions associated with large plastic deformations. With these enhancements, our model is capable of replicating the *in vivo* observations for lateral distraction osteogenesis in sheep using the same differentiation rules and the same set of parameters that successfully describes callus healing in sheep, indicating that tissue differentiation hypotheses originally developed for fracture healing scenarios might indeed be applicable to distraction as well. The response of the model to modified distraction parameters corresponds to existing studies, although the currently available data is insufficient for rigorous validation. As such, this study provides a first step towards developing models that can serve as tools for identifying both interesting research questions and, eventually, even optimizing clinical procedures once better data for calibration and validation becomes available.

## Introduction

Distraction osteogenesis (DO) is a remarkably effective and therefore well-established method for treating skeletal malformations, leg-length discrepancies and large bone defects. First crude forms of DO were introduced at the end of the 19^th^ century and further refined in the following decades, but fell out of favor due to high complication rates and ever improving osteosynthesis devices, which at least partially alleviated the need for DO [1–3]. It was only in the late 1980s that the work of Soviet physician Gavriil A. Ilizarov, who had accidentally discovered the concept of bone segment transport in 1951, was widely disseminated in the western world, leading to a surge of interest in the forgotten method [4–7]. Ilizarov and others further advanced this approach, resulting in more reliable, clinically viable DO procedures [4,8–16].

All forms of DO rely on the fact that bone formation can be triggered by appropriate mechanical stimulation. This mechanical stimulus can be created by slowly pulling apart two segments of bone, typically in small so-called distraction increments once or twice a day. The success of the procedure depends, among other factors, on the appropriate choice of distraction rate and interval: If the distraction is too slow, the segments fuse too early. In the case of too rapid distraction, bone formation may not be able to keep up and/or soft tissue damage, nerve irritation and pain may force a premature termination of the treatment. Another issue with DO is the often extensive treatment time, increasing the risk for complications such as infections or severe pain [4]. Minimizing treatment time further requires the correct choice of fixation, as both overly flexible and overly stiff fixation can prolong maturation time of the newly formed bone [13,17].

Frequently recommended values such as distraction rates of 1 mm/day distributed over two increments per day (i.e. 0.5 mm every 12 hours) ultimately rely on intuition, experience and anecdotal evidence rather than a systematic understanding of the involved processes [4,6,7,13]. Optimal distraction parameters might very well depend on factors such as age, sex, localization and general health status; generic recommendations do not regard any of those factors. A better understanding of the mechanobiolgical processes governing tissue differentiation during DO might therefore provide an opportunity to identify improved procedures that reduce the rate of complications and failure.

Implementing those theories in numerical models allows us to test the validity of the underlying hypotheses by comparing the simulated outcomes with experimental findings. Once we have established a reliable model, it may be useful as a research tool for identifying interesting research questions (*in silico* pilot studies) or, eventually, even aid in optimizing clinical procedures. While the simulation of fracture healing and, more generally, bone healing and remodeling has come a long way since the initial efforts more than 20 years ago, only few numerical models of DO exist.

Carter et al. used a finite element (FE) model to investigate the mechanical stimulation occurring in leg lengthening (mouse model), but only considered a single point in time instead of modeling time-dependent processes, similar to Loboa et al., who investigated mandibular DO [18,19]. Similarly, Morgan et al. simulated the mechanics of a single distraction step for the human tibia, but using a poroelastic material model to describe stress relaxation [20]. Isaksson et al. were, to our knowledge, the first to report about a dynamic model of DO based on the fracture healing model of Lacroix et al., simulating the actual healing process over time [21,22]. Reina-Romo et al. developed models based on the tissue differentiation model by Gómez-Benito et al. and applied them to multiple clinical scenarios [23–26]. Those models, however, do not consider viscoelastic relaxation and assume a stress-free state at the beginning of each distraction step, although studies have shown that this is actually not the case [27–29]. More recently, Reina-Romo et al. therefore improved their model by incorporating ideas from Doblaré and García-Aznar [30,31].

One major obstacle in developing models of DO is that the influence of tensile strain, the major mechanical stimulus in DO, on bone formation is comparatively poorly understood, as most tissue differentiation hypotheses focus on callus healing, where compression is the predominant form of stimulation. Another problem from which many models suffer is that they are based on *in vivo* experiments with highly uncertain, complex loading conditions and mechanical boundary conditions.

**Fig 1:**
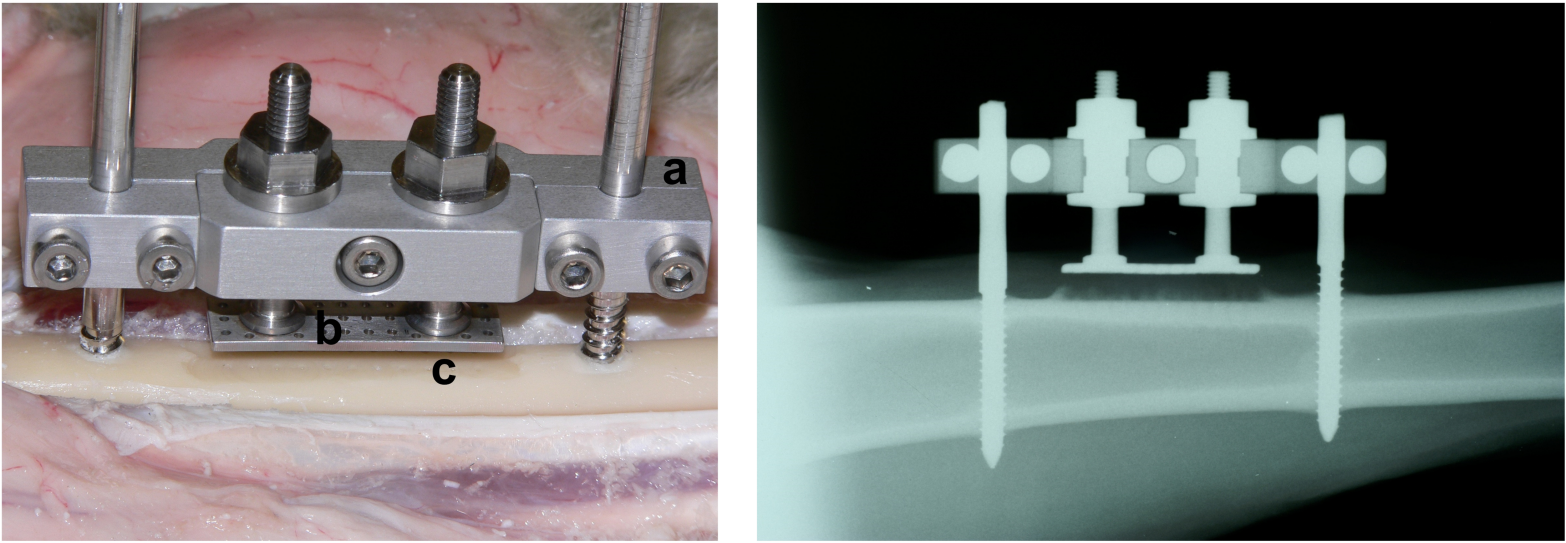
Lateral distraction study by Claes et al. [32]. Left: *In vivo* setup of the study with (a) fixator, (b) hydroxyapatite-coated titanium plate and (c) face milled medial surface of the diaphysis; right: X-ray taken 4 weeks post-op, showing traces of initial bone formation above the medial surface.

The core idea of this study is therefore to develop a new model of DO based on a mechanically simple, well-understood *in vivo* study of lateral distraction in sheep [32]: As described in detail by Claes et al., a hydroxyapatite-coated titanium plate was pulled away from the face milled medial surface (*facies medialis*) of the diaphysis of the right tibia perpendicular to the long axis of the bone (Fig 1). Increments of 0.27 mm twice per day over the course of 10 days resulted in a total transport distance of 5.4 mm. Bone formation started inside the drill holes through the cortex and resulted in the formation of characteristic bony cones. The authors also observed, that bone formation continued after the last distraction step till the end of the experiment despite no further mechanical stimulation, hinting at a previously unknown “memory” effect.

As a joint effort of *in vivo* experiments, mathematical modeling and numerical simulation, this study enables us to gain insight into the peculiarities of DO over “normal” fracture healing. Based on the conceptual understanding derived from the aforementioned *in vivo* experiment, we developed a model that can help us assess the plausibility, validity and applicability of existing tissue differentiation hypotheses to DO and discover systematic relationships between healing success and treatment parameters.

## Modeling distraction osteogenesis

The Ulm tissue-level bone healing model [33–37] rests upon the foundations of Claes & Heigele's quantitative mechanoregulation hypothesis for secondary fracture healing [38]. Local strains induced by external loads are the mechanical stimuli driving the formation and differentiation of the involved tissue types bone, cartilage and connective tissue proper (soft tissue). Biological stimuli such as local and non-local tissue concentrations as well as the level of vascularization complement the mechanical stimuli and further modulate tissue differentiation. Linguistic rules evaluated by a fuzzy logic controller encode the rules governing the differentiation processes.

Our model of distraction osteogenesis is a direct descendant of both this conceptual model as well as its numerical implementation, augmented with some additional features required to manage the peculiarities of distraction osteogenesis. Broadly speaking, our model predicts how the tissue distribution within some considered geometric domain changes over time under the influence of both mechanical and biological stimuli.

**Fig 2:**
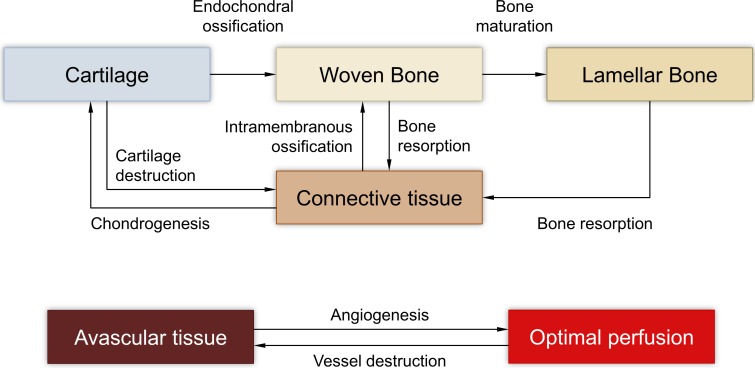
Tissue-level biological processes captured by our model.

The bone healing model captures the following biological processes (Fig 2):

- Intramembranous ossification: Fibrous connective tissue evolves into woven bone
- Endochondral ossification: Fibrocartilage transforms to bone tissue
- Bone maturation: Woven bone is slowly replaced by lamellar bone
- Tissue destruction: Too high mechanical loads may cause the destruction of existing cartilage or bone tissue
- Bone resorption: Existing bone tissue is replaced by soft tissue if the mechanical stimulation falls below a certain threshold.
- (Re-)Vascularization: Initially avascular tissue is revascularized over time.

To describe these processes we represent the current biological state of the simulation domain as a spatiotemporal vector field

$$c:\Omega \times R_{0}^{+}\to {\left[0,1\right]}^{5}$$

$$c:(x,t)\mapsto [c_{w},c_{l},c_{c},c_{s},c_{v}]$$

of relative concentrations *c*_*i*_ ∈ [0,1] of tissue type *i* ∈ {w,l,c,s}, composed of immature (woven) bone *c*_w_ and mature (lamellar) bone *c*_l_, cartilage *c*_c_ and soft tissue *c*_s_, observed in some spatial domain, usually Ω ⊂ ℝ^3^. These tissue components always sum to 1.0 at each point ***x*** of the domain Ω. Soft tissue *c*_s_ acts as a residual/fill up tissue and is thus given implicitly as *c*_s_ = 1.0 − *c*_l_ − *c*_w_ − *c*_c_. As a further state variable vascularity *c*_v_ ∈ [0,1] encodes the relative density of blood vessels with *c*_v_ = 0 in avascular tissue and *c*_v_ = 1 in the case of optimal blood perfusion.

In order to predict the biological state at time *t*_1_ given the initial state ***c***_0_ = ***c***(***x***,*t*_0_) our simulation has to solve the corresponding initial value problem (IVP) by integrating the rate of change over time, i. e. the numerical model approximates

$$c(x,t_{1})=c(x,t_{0})+\int_{t_{0}}^{t_{1}}\partial_{t}c(x,t)dt$$

for each ***x*** ∈ Ω, where ∂_*t*_***c*** refers to the partial derivative of ***c*** with respect to time *t*. The "tissue differentiation function" ***f*** determines the rate of change of the concentration field

$$\partial_{t}c(x,t)=f(b(x,t),m(x,t)),$$

depending on biological stimuli ***b*** and mechanical stimuli ***m***. This system is non-local both in time and space since the biological stimuli ***b*** at each point ***x*** depend on the state within a finite neighborhood (expressed as spatial convolution) and the mechanical stimuli depend on the strain history (expressed as temporal convolution), leading to a system of non-linear delay-integro-PDEs. Solving this problem thus requires (a) determining the current local and non-local mechanical and biological stimuli, (b) estimating how the tissue will react to these stimuli and finally (c) numerically integrating the rates of change over time.

**Fig 3:**
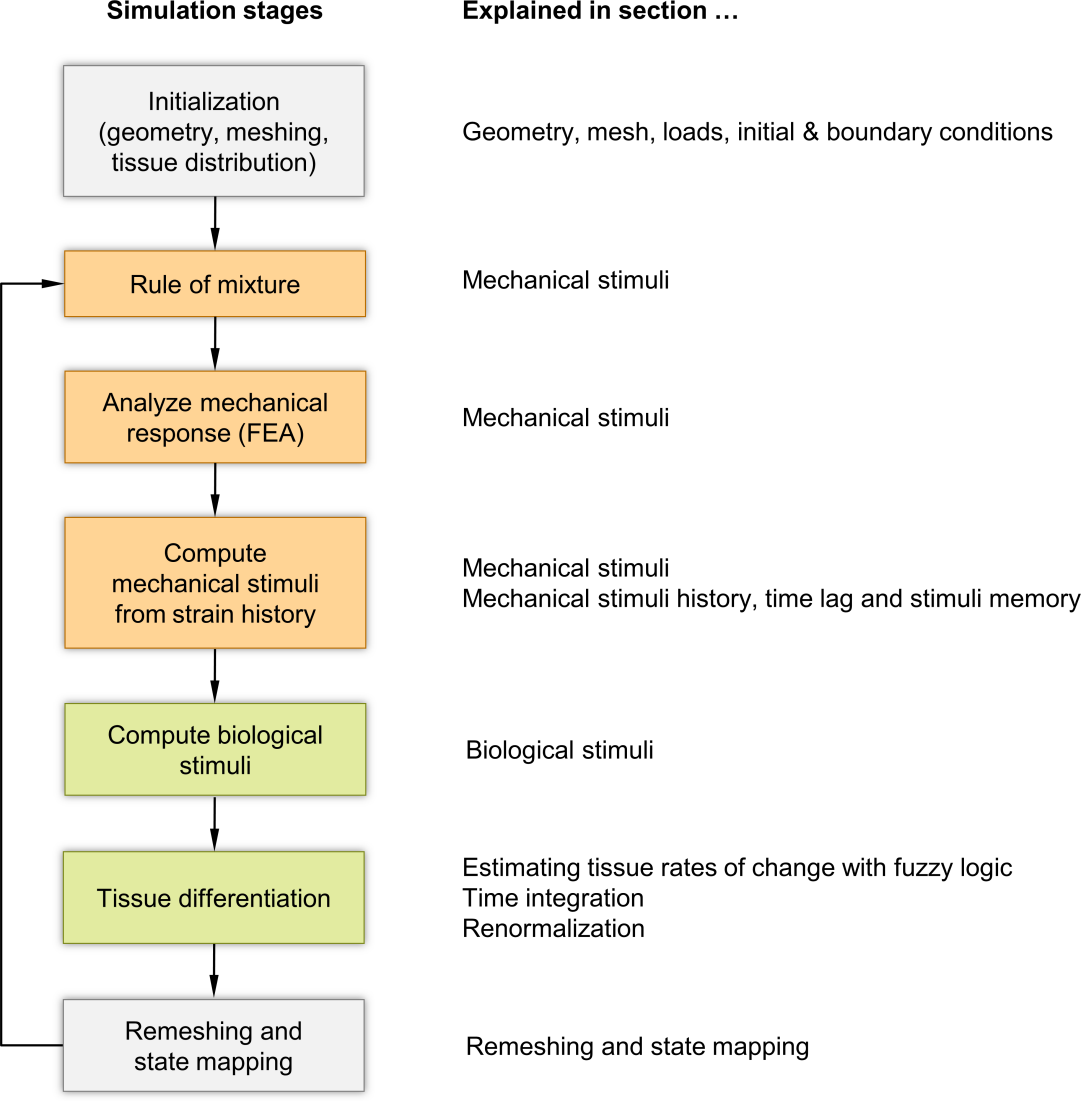
Overview of the simulation phases. The numerical model consists of seven distinct phases (left) that are explained in detail in the corresponding Methods sections (right).

To handle this problem numerically, we subdivide this conceptual model into seven distinct phases (Fig 3): We first define the geometry and the finite element mesh, loads and boundary conditions as well as the initial tissue distribution. Then we enter a loop, which consists of determining the mechanical and biological stimuli and—based on these stimuli—changing the tissue concentrations accordingly. Before entering the next iteration, we remesh the domain and transfer the mechanical and biological state to the new mesh. The following sections explain each of those phases in detail.

### Geometry, mesh, loads, initial & boundary conditions

**Fig 4:**
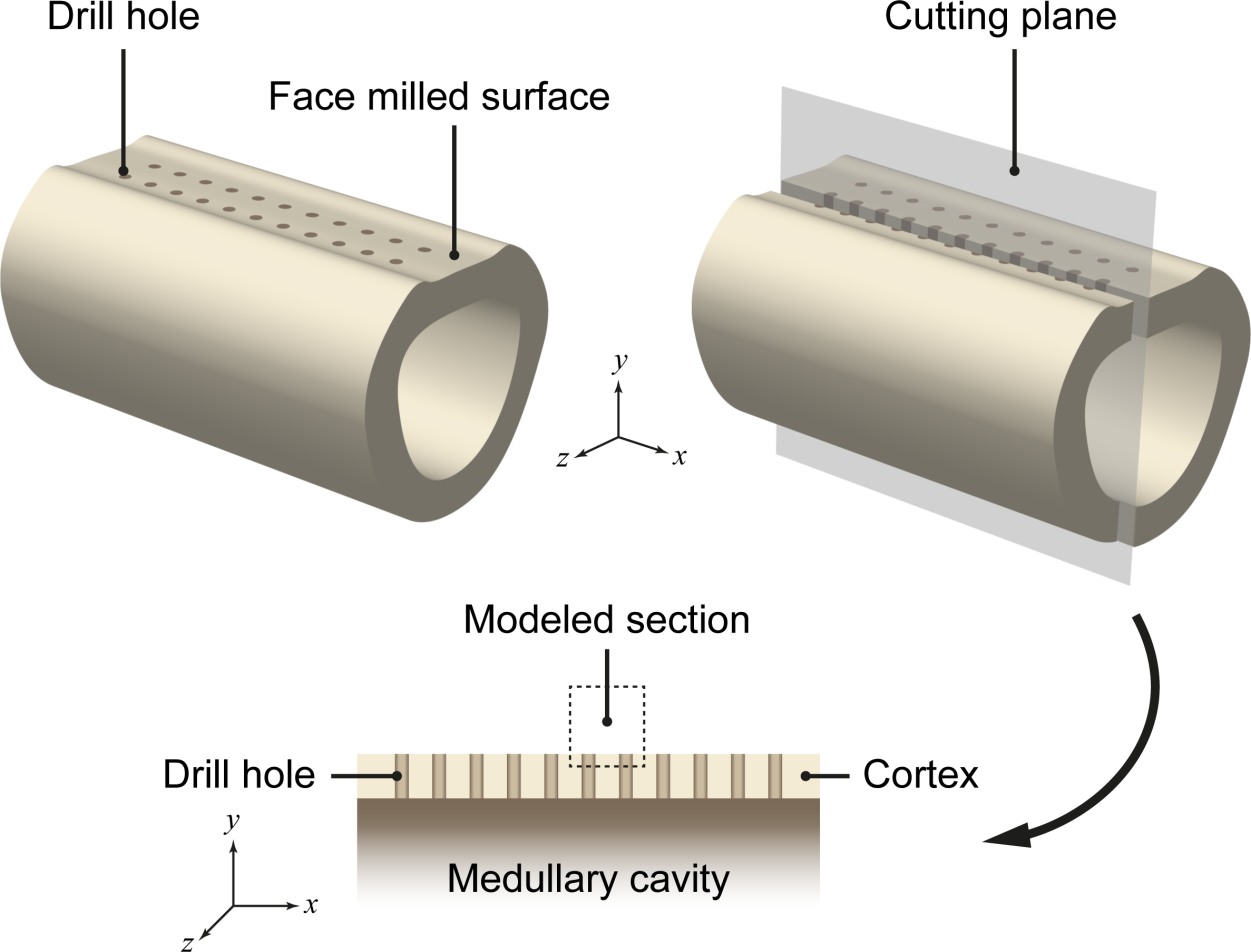
The model’s 2D geometry corresponds to a slice through one row of the drill holes [37].

**Fig 5:**
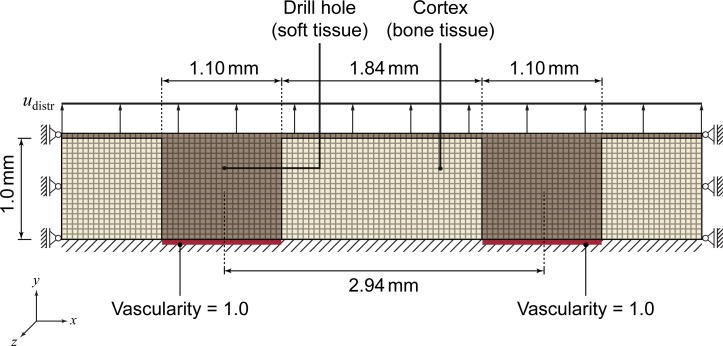
Geometry and FE mesh, mechanical load and boundary conditions as well as initial tissue distribution [37].

To reduce the computational complexity for this particular case, we simplified the problem to a 2D approximation by only considering the middle section of a thin slice (plane stress assumption) through one row of the drill holes (Fig 4). The resulting 2D geometry covers two of the 22 drill holes, a 1.0 mm thin cortical layer and a 0.05 mm = one layer of finite elements) thin layer of connective tissue (Fig 5). Because the periosteum was removed during surgery, we assume the whole region to initially be avascular (*c*_v_ = 0). Suitable boundary conditions for vascularity (*c*_v_ ≡ 1) enable revascularization via the drill holes and hence from within the medullary canal. The drill holes are initially filled with connective tissue (*c*_s_ = 1), while the cortex consists of lamellar bone (*c*_l_ = 1).

We apply no-displacement Dirichlet boundary conditions to the bottom of the cortex layer by fixing all mechanical DOFs (*u*_*x*_ ≡ *u*_*y*_ ≡ 0). The left and right domain borders are fixed in one DOF (*u*_*x*_ ≡ 0, *u*_*y*_ free), assuming periodicity. During the distraction phase we apply the distraction load *u*_distr_ (displacement in *y* direction) at the top of the connective tissue layer in a stepwise fashion, e. g. *u*_distr_ = 0.27 mm every *t*_distr_ = 12 hours in case of the original in vivo protocol. To simulate both the post-operative latency period as well as the consolidation phase, the top of the connective tissue layer is fixed in vertical direction (*u*_*y*_ ≡ 0).

### Mechanical stimuli

We assume that tissue differentiation is, first and foremost, guided by mechanical stimulation. Following Pauwels, we use two invariants of the strain tensor to characterize the local strain state and decide the fate of the tissue at each point [33,34,39]: Distortional strain

$$\gamma =\frac{1}{\sqrt{2}}\sqrt{{\left(\varepsilon_{1}-\varepsilon_{2}\right)}^{2}+{\left(\varepsilon_{1}-\varepsilon_{3}\right)}^{2}+{\left(\varepsilon_{2}-\varepsilon_{3}\right)}^{2}}$$

with principal strains *ε*_1_, *ε*_2_ and *ε*_3_ quantifies the amount of pure shape change and is closely related to octahedral shear strain. Dilatational strain

$$\varepsilon =\frac{1}{3}(\varepsilon_{1}+\varepsilon_{2}+\varepsilon_{3})$$

on the other hand describes the pure volume changing fraction of the total strain. In accordance with Frost's “mechanostat” hypothesis as well as Claes & Heigele's tissue differentiation hypothesis, we assume that bone as a mechanosensitive tissue adapts itself in such a way via modeling/remodeling that mechanical strain is kept within a preferred equilibrium range [38,40,41].

Since the material properties at each point depend on the local tissue composition, we use a rule of mixture (RoM) [34,42] to derive composite linear elastic material properties from the concentration field with Young’s modulus

$$E(x,t)=\sum_{i}E_{i}c_{i}{\left(x,t\right)}^{3}\;\mathrm{where}\;i\in \{w,l,c,s\}$$

and Poisson’s ratio

$$\nu (x,t)=\sum_{i}\nu_{i}c_{i}(x,t).$$

The elastic properties *E*_*i*_ and *ν*_*i*_ of the pure materials match that of the fracture healing model [33] (Table 1). In the numerical model, we sample the tissue concentration field at element centroids of the FE mesh and derive the material properties for each element from these samples according to the rule of mixture above.

**Table 1:** Linear elastic material properties of tissue types.

Tissue *i*	Young’s modulus *E*_*i*_ (MPa)	Poisson’s ratio *ν*_*i*_
Lamellar bone	10,000	0.3
Woven bone	4,000	0.3
Fibrocartilage	200	0.4
Connective tissue	3	0.3

In our previous fracture healing simulations, we could use static structural FEAs to derive the characteristic mechanical stimuli for each time step. Biological tissues under distraction loads, however, exhibit time-dependent (viscoelastic) behavior [28,29,43]. After applying a distraction load step, the stresses induced by the distraction load relax at an exponential rate. Beyond a certain yield stress, permanent deformation may develop (e. g. rupturing of collagen fibers) as well.

In order to describe the evolution of such a time-dependent strain field we added viscoplastic material properties on top of the baseline linear elastic behavior. Exact parameter values required to describe the visco-elastoplastic behavior are however largely unknown and we can only estimate approximate parameters from indirect measurements [43, 44]. Because of that and in order to avoid increasing the number of unknown parameters much further, we chose the Perzyna model as it is the most simple viscoplastic material model ANSYS has to offer [45]. According to this model, von Mises (equivalent) viscoplastic strain rate ${\dot{\varepsilon }}_{\mathrm{vp},\;\mathrm{eqv}}$ is related to von Mises (equivalent) stress *σ*_eqv_ as described by the following constitutive equation:

$${\dot{\varepsilon }}_{\mathrm{vp},\;\mathrm{eqv}}=\gamma {\left(\frac{\sigma_{\mathrm{eqv}}}{\sigma_{\mathrm{yield}}}-1\right)}^{\frac{1}{m}}\;\mathrm{for}\;\sigma_{\mathrm{eqv}}>\sigma_{\mathrm{yield}}$$

Based on the limited available literature data, we estimated the viscoplastic material parameters *γ*, *σ*_yield_ and *m* as well as *E*_T_ for bilinear isotropic hardening as given by Table 2 to approximately fit the empirically determined relaxation behavior.

**Table 2:** Viscoplastic material properties relative to the composite Young’s modulus *E*.

Parameter	Estimated value
Fluidity *γ*	10^−6^ s^−1^
Strain rate hardening *m*	1.0
Static yield stress *σ*_yield_	10^−3^ *E*
Tangent modulus *E*_T_	10^−2^ *E*

To compute the time-dependent strain field we have to perform a non-linear, transient FEA for each distraction step, consisting of applying the required distraction displacement and subsequent stress relaxation. Furthermore, we need to derive characteristic mechanical stimuli that drive the differentiation process from the now time-dependent strain signal. An approach resembling that of our fracture healing simulations is to sample only the peak strains within each distraction cycle, assuming that singular (or rather: few) peaks of mechanical stimulations each day completely determine tissue differentiation, i. e. we will be using the peak-stimuli signals

$$\gamma_{\mathrm{peak}}(t)=\max\left\{\gamma (\tau )|t-t_{distr}\leq \tau \leq t\right\}$$

and

$$\varepsilon_{\mathrm{peak}}(t)=\max\left\{\varepsilon (\tau )|t-t_{distr}\leq \tau \leq t\right\}.$$

One might think of this sampling strategy as a crude approximation of a potential non-linear response of the mechanosensory cells to mechanical stimulation, where high strains mask following minor stimuli.

### Mechanical stimuli history, time lag and stimuli memory

According to the aforementioned tissue differentiation hypothesis, tissue reacts instantaneously to any change in mechanical stimulation. Obviously, this is a severe simplification of reality: The strain-sensing cells need to convert mechanical signals to biochemical ones and the targeted cells in turn need time to react to this stimulation (proliferation, migration, differentiation etc.). New extracellular matrix (ECM) must be synthesized and requires some time to calcify. Mechanical stimulation merely triggers this cascade of biological processes, which eventually will lead to a visible change in calcified tissue concentration. A second, related effect is that osteogenesis continues even after all mechanical stimulation, including residual stresses/elastic strains, have faded away [32]. The biological processes underlying tissue differentiation, and in particular osteogenesis, seem to possess some kind of “memory” for past mechanical stimulation [46].

Our model captures both of these effects on osteogenesis by folding the history of mechanical stimuli with an appropriate “memory kernel”: We simulate the time lag between cause (mechanical stimulation) and effect (change in bone tissue concentration) by delaying the strain signal we feed into the differentiation function by an amount of *t*_delay_. To approximate the influence of the stimuli memory, we weight past stimuli with an exponentially decaying weight function. The resulting combined convolution kernel is

$$w(t)=\left\{ \begin{array}{ll} \exp\left(-\lambda_{\mathrm{decay}}(t-t_{\mathrm{delay}})\right) & \mathrm{if}\;t_{\mathrm{delay}}<t\leq t_{\mathrm{mem}}\\ 0 & \mathrm{otherwise} \end{array}\right.$$

and its normalized form

$$\hat{w}(t)=\frac{w(t)}{\int_{-\infty }^{+\infty }w(\tau )d\tau }$$

The parameter *λ*_decay_ controls how quickly the influence of past stimuli decay. We consider stimuli older than *t*_mem_ to have no influence on tissue differentiation. With the convolution of two real-valued functions *f* and *g* defined as

$$(f*g)(t)≔\int_{-\infty }^{\infty }f(\tau )g(t-\tau )d\tau$$

we can now express the effective mechanical stimuli that drive osteogenesis as

$$\gamma_{\mathrm{eff}}=\gamma_{\mathrm{peak}}*\hat{w}$$

$$\varepsilon_{\mathrm{eff}}=\varepsilon_{\mathrm{peak}}*\hat{w}.$$

The mechanical stimulation is therefore fully specified by the vector

$$m=[\gamma_{\mathrm{peak}},\varepsilon_{\mathrm{peak}},\gamma_{\mathrm{eff}},\varepsilon_{\mathrm{eff}}].$$

Note that, as tissue differentiation now also depends on *past* stimuli, formally, we have to solve a system of *delay* partial differential equations instead of "ordinary" PDEs. This also further implies that, instead of instantaneous initial conditions only, we also will have to specify an initial stimuli *history*. Thus ***m*** not only depends on ***x*** and *t*, but really on the *entire concentration field* ***c*** up to time *t*, both spatially (solving the mechanical BVP at *t* requires ***c***(⋅,*t*) to derive the material properties for the whole spatial domain) and temporally (*ε*_eff_ and *γ*_eff_ both depend on the mechanical stimuli history *ε* and *γ* up to *t* and therefore indirectly on ***c*** as well). Formally ***m*** is a functional M

$$m(x,t)=M(x,t,c(\cdot ,t_{0}\ldots t),u_{BC},F)$$

with mechanical displacement boundary conditions ***u***_BC_ and applied external forces ***F*** (please refer to S2 File for a definition of the mathematical notation used throughout the manuscript).

### Biological stimuli

While mechanical stimulation is a critical prerequisite for bone formation, nutrient supply is just as important [47]. The model therefore considers (relative) blood vessel density, which we call “vascularity” = component *c*_v_) and which we assume to be strongly correlated with nutrient and oxygen supply. Our differentiation rules (see S1 File) therefore require a certain threshold vascularity for osteogenesis.

The local biological environment, represented in the model by the concentration vector ***c***, controls the differentiation path: For instance, cartilage is a prerequisite for endochondral ossification, as opposed to intramembranous ossification, which will only occur in connective tissue. Likewise, bone resorption as well as maturation can only take place in ossified regions.

Assuming that osteocytes sense mechanical stimulation and summon nearby osteoblasts (lining cells, precursors) via paracrine signaling to commence osteogenesis, bone formation should only be possible in the proximity of already existing bone tissue. We capture this influence of adjacent tissue on the local differentiation process by using the distance-weighted average bone tissue concentration as an indicator for the relative strength of biochemical stimulation *s*_b_ generated by nearby osteocytes [48,49]; more formally

$$s_{b}(x,t)=\left((c_{w}(\cdot ,t)+c_{l}(\cdot ,t))*G_{\mathrm{adj}}\right)(x)$$

with a Gaussian spatial convolution kernel (here in 2 dimensions)

$$G_{\mathrm{adj}}(x,y)=\frac{1}{2\pi \sigma_{\mathrm{adj}}^{2}}\exp\frac{-x^{2}-y^{2}}{2\sigma_{\mathrm{adj}}^{2}}.$$

The actual discretized version of *G*_adj_ implemented is normalized to ensure that the sum of all weights equals one and hence convolving the bone concentration with the normalized kernel yields the weighted arithmetic mean.

*G*_adj_ defines an *L*^2^ distance-weighted, finite "region of influence", representing the paracrine signaling range and its distance-dependent relative strength. We chose *G*_adj_ (the “heat kernel”) specifically to approximate diffusion of growth factors into the surrounding area. Osteogenesis can only occur in places, where *s*_b_ exceeds a given threshold value (S1 File). Note that this model does not explicitly capture the entire cascade of how signaling molecules are produced, diffuse and are absorbed by their target cells, which in turn react to that signal. Instead, we assume that the time lag *t*_delay_ introduced by folding the mechanical stimuli with $\hat{w}$ approximately accounts for the necessary delay between cause (stimulation) and effect (bone formation).

Similarly to osteogenesis, both sprouting and splitting angiogenesis require existing vasculature. Analogous to *s*_b_ for osteogenesis, we therefore define

$$s_{v}(x,t)=(c_{v}(\cdot ,t)*G_{\mathrm{adj}})(x)$$

to be the (distance-weighted) average vascularity, modelling the influence of the vasculature and its nutrient supply on tissue differentiation and revascularization.

The biological part of the stimuli fed into the tissue differentiation function ***f*** is thus

$$b=[c_{w},c_{l},c_{c},c_{s},c_{v},s_{b},s_{v}].$$

Analogous to the mechanical stimuli, we can make the dependencies of ***b*** more explicit by stating that

$$b(x,t)=B(x,t,c(\cdot ,t),c_{BC}),$$

meaning that the biological stimuli depend on the current concentration field and some concentration boundary conditions ***c***_BC_.

### Estimating tissue rates of change with fuzzy logic

The tissue differentiation function ***f*** governing the tissue differentiation process is formulated in terms of linguistic rules and associated membership functions, forming a knowledge base for a Mamdani-type fuzzy inference system [50,51]. By evaluating the linguistic rules, the fuzzy logic controller maps the previously computed biological stimuli ***b*** and mechanical stimuli ***m*** to concentration rates of change Δ*c*_w_,Δ*c*_c_,Δ*c*_v_ (in units of day^−1^) for woven bone, cartilage and vascularity.

The rules and membership functions are largely based on those previously introduced by Simon et al. for secondary fracture healing (see S1 File). Bone formation, however, now depends on effective (i. e. memorized and delayed) stimuli instead of the instantaneous strain signal. Furthermore, we replaced the input variables “maximum adjacent bone concentration” and “maximum adjacent vascularity” with *s*_b_ and *s*_v_, respectively.

We also added a rule to facilitate the simulation of bone resorption, removing superfluous bone material in under-stimulated tissue. The actual woven bone resorption rate *r*_w_ depends on the degree of under-stimulation ∈ [0,1] determined by evaluating the aforementioned rule, scaled by the maximum woven bone resorption rate, defined as

$$r_{w}^{\max}=\rho_{w}g_{w}^{\max}$$

where the factor *ρ*_w_ relates the maximum resorption rate to the maximum formation rate of woven bone $g_{w}^{\max}=18\%/\mathrm{day}$ using the differentiation rules documented in S1 File. The resorption rate of lamellar bone, on the other hand, is limited by

$$r_{l}^{\max}=\rho_{l}g_{w}^{\max}.$$

As (compact) lamellar bone is less porous than woven bone and thus has a lower surface area density, resorption of lamellar bone should proceed slower than in woven bone. Our model can account for this effect by choosing a lower value for *ρ*_l_ compared to *ρ*_w_. By default, we chose a resorption rate ratio of ρlρw=14 [52–54]. While this formulation works well for callus healing [37], we had to disable bone resorption for the simulation of lateral distraction osteogenesis in order to avoid complete resorption during the consolidation phase (see Discussion).

Over the course of the consolidation period, much stiffer mature lamellar bone slowly replaces the initial immature (woven) bone. In our model, maturation does not require any particular stimulus. Instead, new lamellar bone replaces existing woven bone at a constant rate of $g_{\mathrm{mat}}=0.1\cdot g_{w}^{\max}=1.8\%/\mathrm{day}$, resulting in a propagation velocity of the bone front of roughly 3–4 μm/day [52].

The final concentration rates of change after the described differentiation, resorption and maturation procedures therefore are

$$\Delta c=[\Delta c_{w},\Delta c_{l},\Delta c_{c},\Delta c_{s},\Delta c_{v}]$$

with

$$\Delta c_{s}=-\Delta c_{w}-\Delta c_{l}-\Delta c_{c}$$

following from the definition of *c*_s_.

### Time integration

Being a comparatively slow process, the effect of tissue remodeling on the mechanics over the course of a sufficiently small time step is negligible. A transient finite element analysis per distraction step with fixed material properties therefore determines the time-dependent strain field driving tissue differentiation. This method decouples the inner BVP from solving the overall IVP (method of lines).

We use a simple explicit Euler scheme to integrate the concentration rates of change over time. While originally the step size was fixed at Δ*t* = 1 day [33,34], simulating different distraction osteogenesis protocols requires a more flexible time integration scheme with an adjustable time step size Δ*t* in order to cope with varying distraction intervals *t*_distr_ = *n* ⋅ Δ*t* with *n* ∈ ℕ. With forward Euler this leads to an approximate solution of the form

c(x,t+Δt)≈c(x,t)+ΔtΔc,

requiring *n* time-integration steps per distraction step.

### Renormalization

There is no way to enforce the concentration constraints ∑_*i*_ *c*_*i*_ = 1 and *c*_*i*_ ∈ [0,1] during the evaluation of the tissue differentiation rules using the fuzzy logic controller provided by Matlab (R2016b, The MathWorks, Inc., Natick, MA). We therefore renormalize the concentrations *c*_*i*_ at the end of each time integration step by

- limiting vascularity to $c_{v}^{\prime }=\min\left\{c_{v},1.0\right\}$,
- cutting off negative concentration values, i. e. $c_{i}^{\prime }=\max\left\{0.0,c_{i}\right\}$,
- and scaling bone and cartilage concentrations by a factor of *κ* = (*c*_w_ + *c*_l_ + *c*_c_)^−1^, but only if *c*_w_ + *c*_l_ + *c*_c_ > 1.0. This effectively projects the concentration vector back onto the plane {(*c*_w_,*c*_l_,*c*_c_) | *c*_w_ + *c*_l_ + *c*_c_ = 1.0} while maintaining the bone/cartilage ratio.

### Remeshing and state mapping

Distraction osteogenesis displaces tissue by several centimeters. Large amounts of plastic strain would lead to severe mesh distortions, rendering the original FE mesh unusable within only a couple of distraction steps. Remeshing the domain after each distraction step avoids this problem, but also necessitates a way to transfer the biological and mechanical state (persisting elastic strains) to the new mesh.

**Fig 6:**
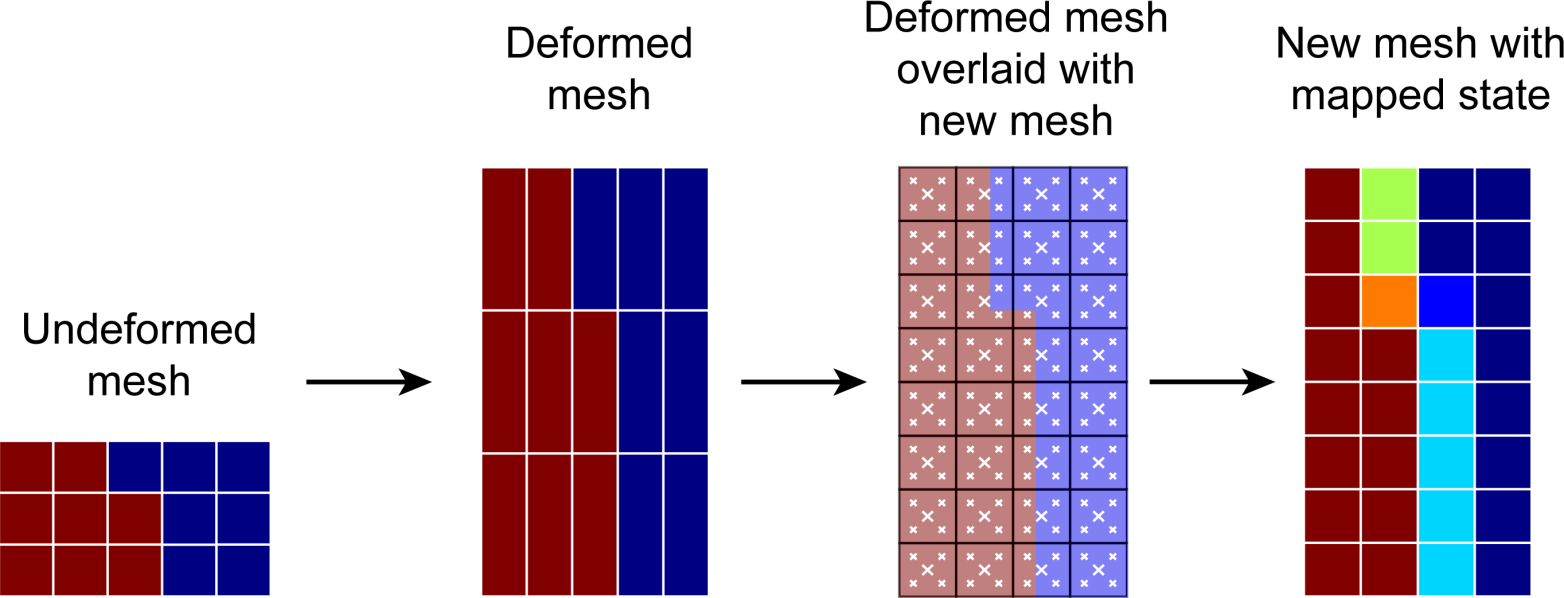
Illustration of the remeshing procedure on a simplified geometry. After remeshing, the state (concentrations, elastic strains) is sampled (white crosses) and mapped to the new mesh [37].

Our method first creates an entirely new mesh based on the geometry at the end of the current distraction step, including all deformation that has occurred so far. We then sample the old mesh at locations corresponding to the centroids of the newly generated elements as well as at four additional points halfway between the centroids and the elements’ corner nodes and assign the weighted average state (concentrations and elastic strain) to the centroid of the new mesh. This method of directly sampling the elementwise-constant tissue distribution represented by the old mesh is a form of rasterization combined with 5 × super-sampling for reducing aliasing artifacts. In contrast to sampling from an interpolated concentration field, this approach is able to preserve sharp material interfaces as accurately as possible (Fig 6).

## Simulations

Compared to the callus healing model, we have introduced some new concepts involving new, unknown parameters, in particular regarding the viscoplastic material properties, the stimuli memory and the non-local influence of biological stimuli (“paracrine signaling”). Unless stated otherwise, the following simulations use the default parameter values given by Table 1, Table 2 and Table 3.

**Table 3:** Default parameter values.

Parameter	Symbol	Value
Time steps per distraction step	*n*	16
Time step size	Δ*t*	*t*_distr_/*n* = 0.75 hours
Spatial resolution*	Δ*x*	50 μm
Stimuli delay	*t*_delay_	5 days
Stimuli memory capacity	*t*_mem_	45 days
Stimuli memory decay rate	*λ*_decay_	0.1/day
Paracrine signaling influence	*σ*_adj_	0.30 mm
Paracrine signaling range	*r*_adj_	0.70 mm = 7/3 *σ*_adj_
Distraction increment	*u*_distr_	0.27 mm
Distraction interval	*t*_distr_	12 hours
Post-operative latency	–	10 days
Distraction phase length	–	10 days
Consolidation time	–	50 days

*edge length of a quadrilateral finite element

### Comparison with *in vivo* results

**Fig 7:**
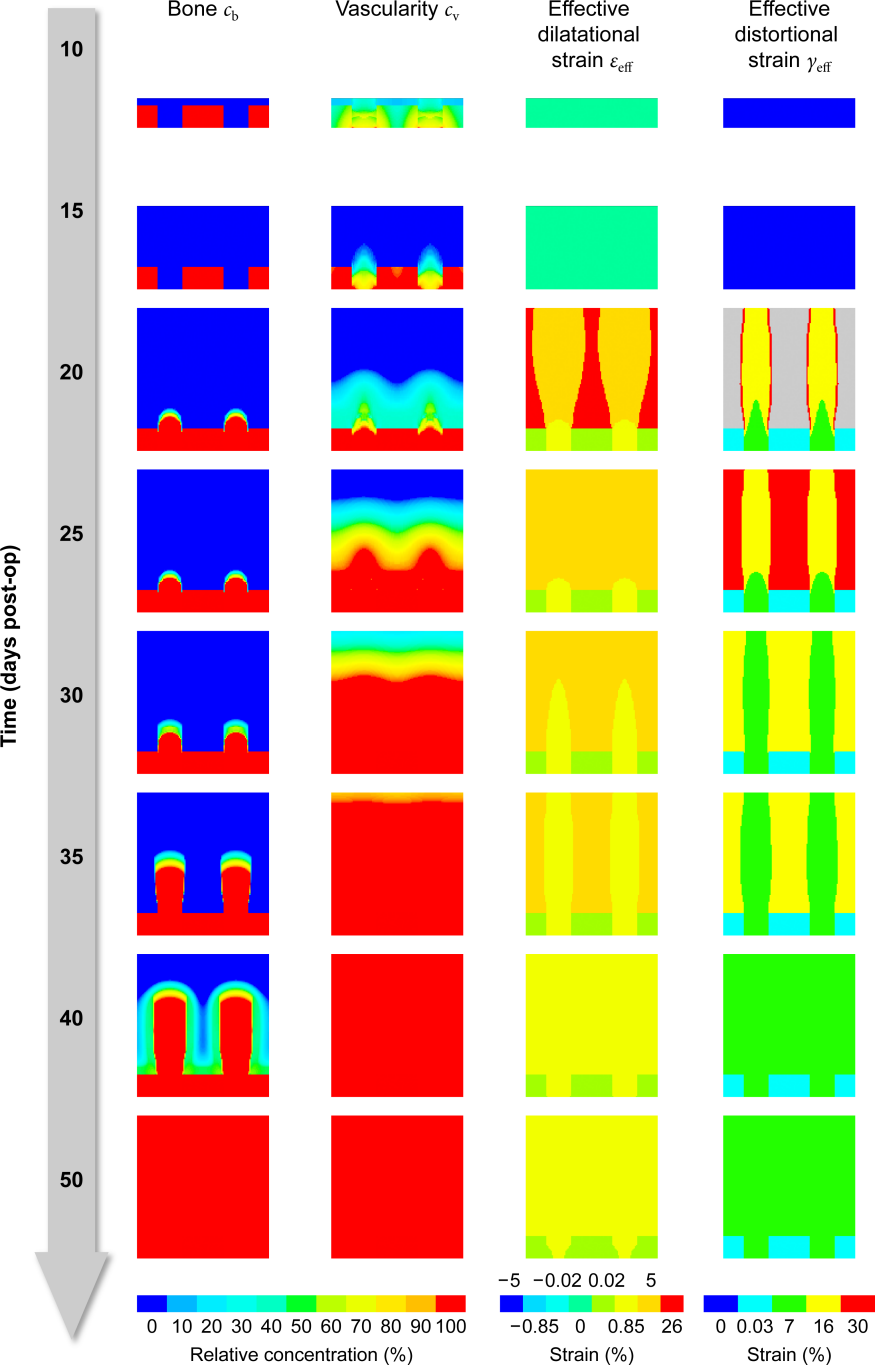
Predicted bone concentration, vascularity and effective mechanical stimuli for the *in vivo* experiment by Claes et al. [32]. The figure shows (from left to right) how the distribution of bone, vascularity and effective dilatational and distortional strain inside the healing area changes over time (from top to bottom). The legends beneath the columns explain the meaning of the color-coding for the corresponding column(s).

Using these carefully tuned parameters, the model is able to reproduce the overall findings of the *in vivo* study (Fig 7): When the distraction phase starts at day 10, angiogenesis has already started to revascularize the healing area. During the distraction phase, angiogenesis is mostly limited to the cortical layer and area inside and slightly above the drill holes, where the mechanical strain is comparatively low. Towards the end of the distraction phase (around day 17) strains become low enough to allow the gradual revascularization of the entire healing region (day 35).

As osteogenesis requires both sufficient blood supply and mechanical stimulation, new bone cannot by observed until day 15, when the first traces of woven bone appear on the inside of the drill holes. Starting from there, bony cones start to grow vertically along a corridor of relatively moderate (effective) mechanical stimulation. Bone growth continues past the last distraction step on day 20 until day 50, when the entire area has been filled with woven bone.

Based on fluorescence labeling with calcein green on day 20 and tetracyclin on day 30, the authors of the *in vivo* study were able to determine the approximate average height of the bony cones to be ≈ 1 mm and ≈ 2 mm, respectively, both of which correspond well to the simulation results. The model predicts an average bone apposition rate of ≈ 100 μm/day, which also falls into the experimentally determined range of 92–110 μm/day.

### Parameter identification & sensitivity analysis

We will now investigate how changing parameters influences the predictions in order to determine the quality of the numerical discretization (convergence analysis), the sensitivity of the model to uncertain parameters (sensitivity analysis), plausible values for unknown parameters (parameter identification) and the potential impact of changing the distraction protocol.

#### Temporal discretization

**Fig 8:**
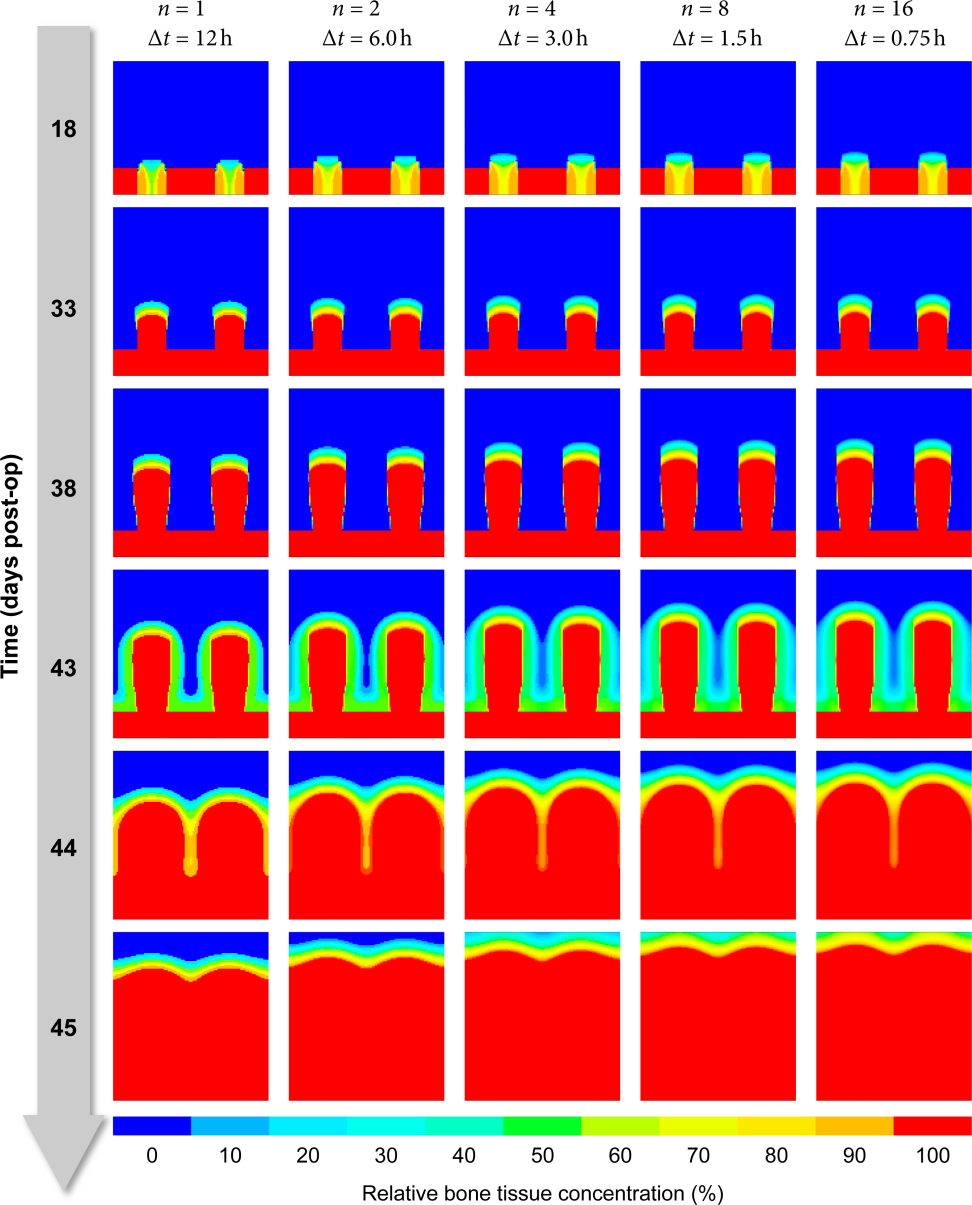
Influence of time step size on predicted bone tissue distribution. Each column displays the evolution of bone tissue over time (rows, top to bottom) from blue (0% bone) to red (100% mineralized bone) for five different temporal discretizations (columns, left to right).

To ensure that the chosen temporal resolution (time step size) suffices and the model predictions converge with increasing resolution, we ran the simulation with *n* = 1,2,4,8,16 time steps per distraction cycle, resulting in time step sizes of Δ*t* = 12,6,3,1.5,0.75 hours (Fig 8). While decreasing Δ*t* from 12 to 6 hours to 3 hours has a clearly visible effect on the tissue transformation rate, further decreasing Δ*t* has only a minuscule effect.

#### Spatial discretization

**Fig 9:**
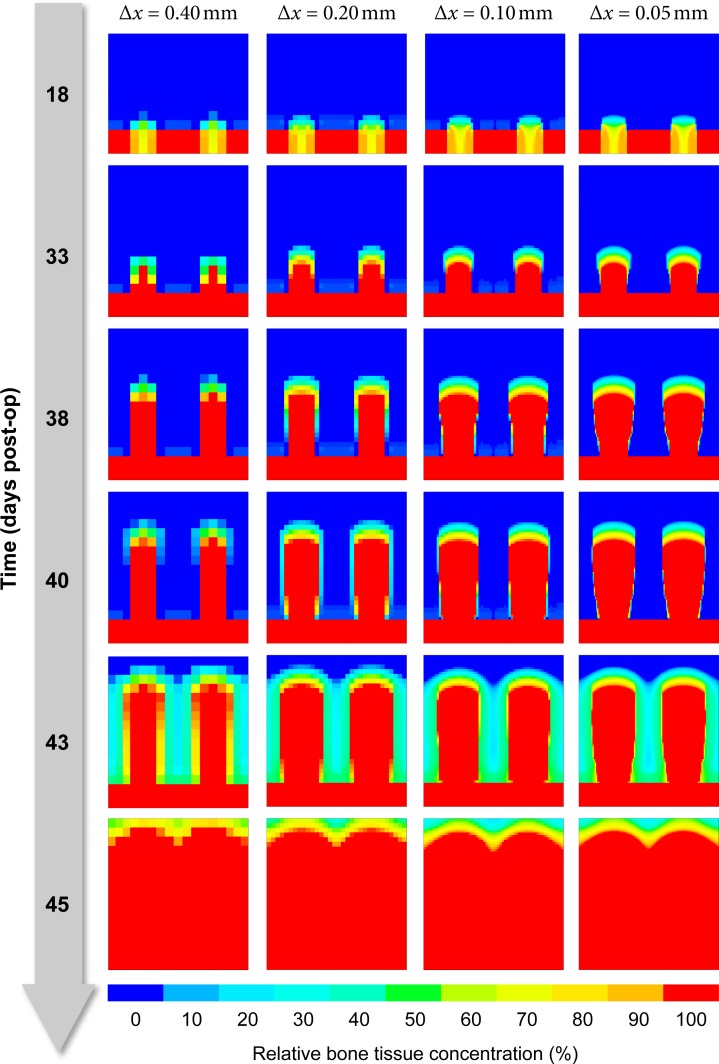
Influence of element size on predicted bone tissue distribution. Each column displays the evolution of bone tissue over time (rows, top to bottom) from blue (0% bone) to red (100% mineralized bone) for four different FE mesh resolutions (columns, left to right).

The resolution of the FE mesh can affect the predicted healing patterns both directly (undersampling the concentration fields) or indirectly due to insufficient accuracy of the mechanical FEA and thus inaccurate mechanical stimuli. Using different element edge lengths of Δ*x* = 0.05,0.10,0.20,0.40 mm however has only a minor effect on the predicted bone tissue distribution (Fig 9). In particular, the propagation speed of the bone front is virtually identical for all four cases, although the highest resolution grid (Δ*x* = 0.05 mm) is able to display fine details more accurately.

#### Material parameters for viscoplasticity

As the viscoplastic properties given in Table 2 are merely a rough estimate chosen to fit the global relaxation behavior and are not backed by actual measurements, the true values may substantially deviate from the assumed defaults. We therefore simulated the distraction procedure with parameter values for *γ*, *E*_T_ and *σ*_yield_ deviating ± one order of magnitude from the defaults to make sure, that the simulation result does not depend strongly on those highly uncertain parameters.

**Fig 10:**
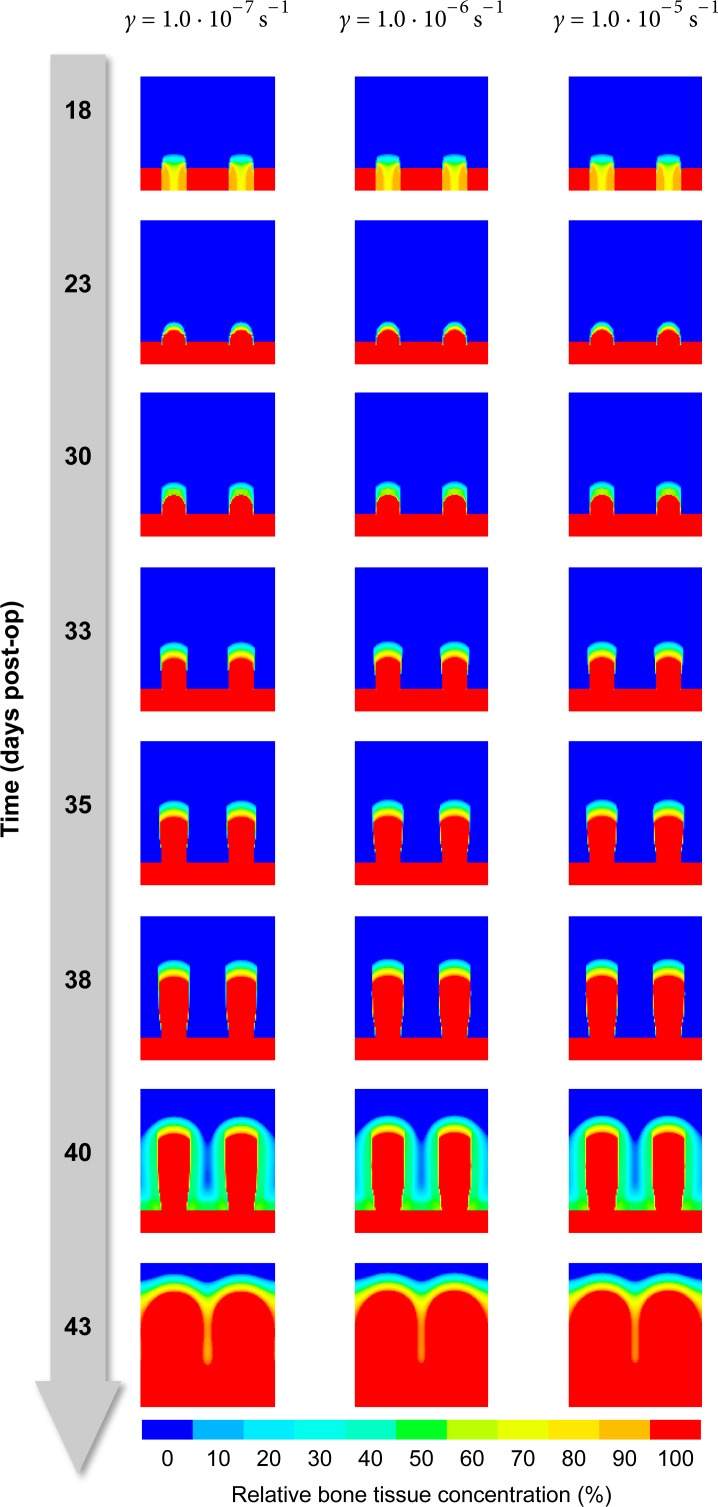
Influence of fluidity. The figure shows how three choices for the parameter *γ* (columns) affects the predicted distribution of bone over time (rows, top to bottom).

**Fig 11:**
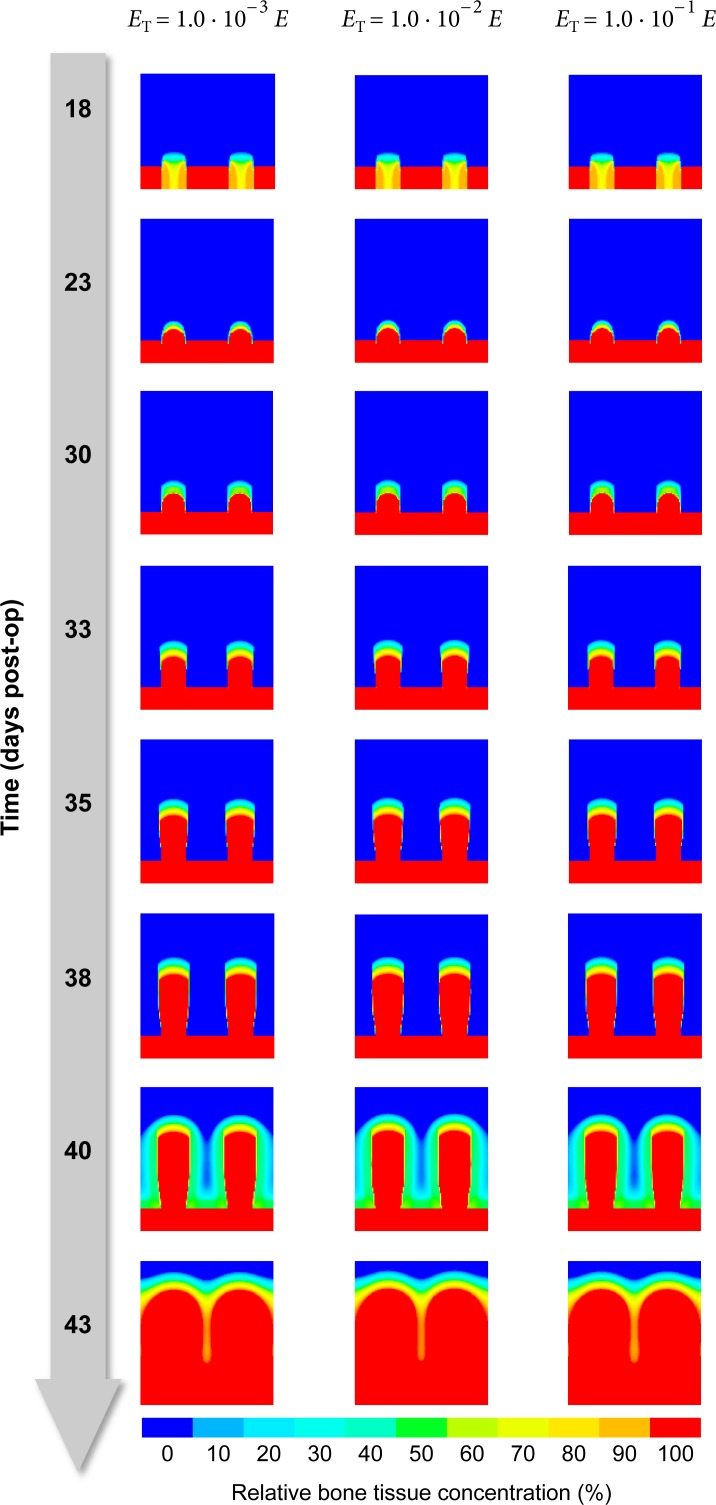
Influence of tangent modulus *E*_T_. The figure illustrates the influence of the tangent modulus *E*_T_ (columns) on the predicted distribution of bone over time (rows, top to bottom).

**Fig 12:**
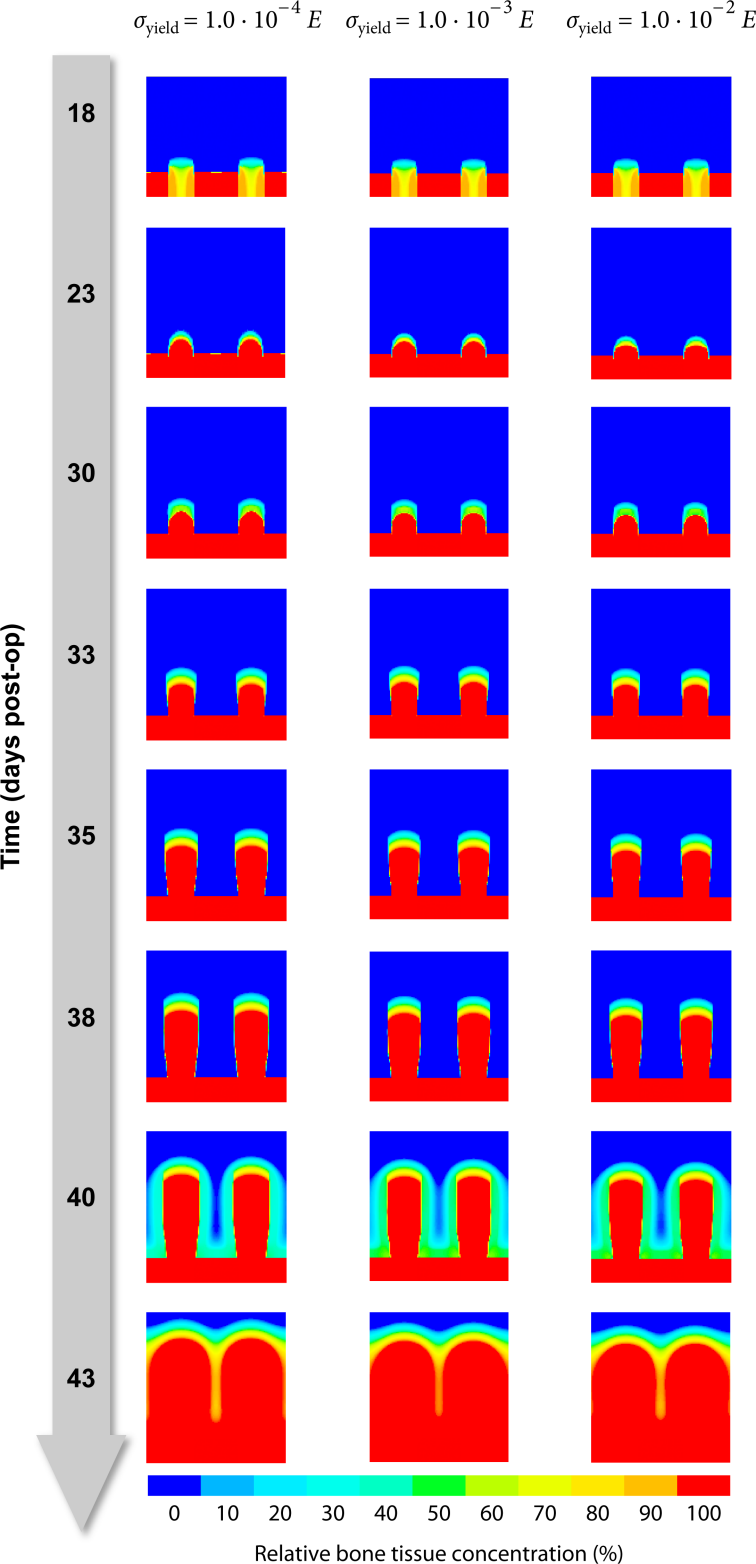
Influence of yield stress *σ*_yield_. Each column represents one particular parameter value for the yield stress parameter *σ*_yield_. The contour plots represent the corresponding predicted healing outcome in terms of the evolution of bone tissue inside the modeled healing region (from top to bottom).

The fluidity parameter *γ* determines how quickly stresses relax and elastic strains transform into plastic deformations. Whether we choose *γ* to be 10^−7^s^−1^, 10^−6^s^−1^ or 10^−5^s^−1^, it does not seem to influence the predicted osteogenesis pattern, though (Fig 10). The same diagnosis applies to varying the tangent modulus *E*_T_ and the yield stress *σ*_yield_ as well (Fig 11 and Fig 12).

**Fig 13:**
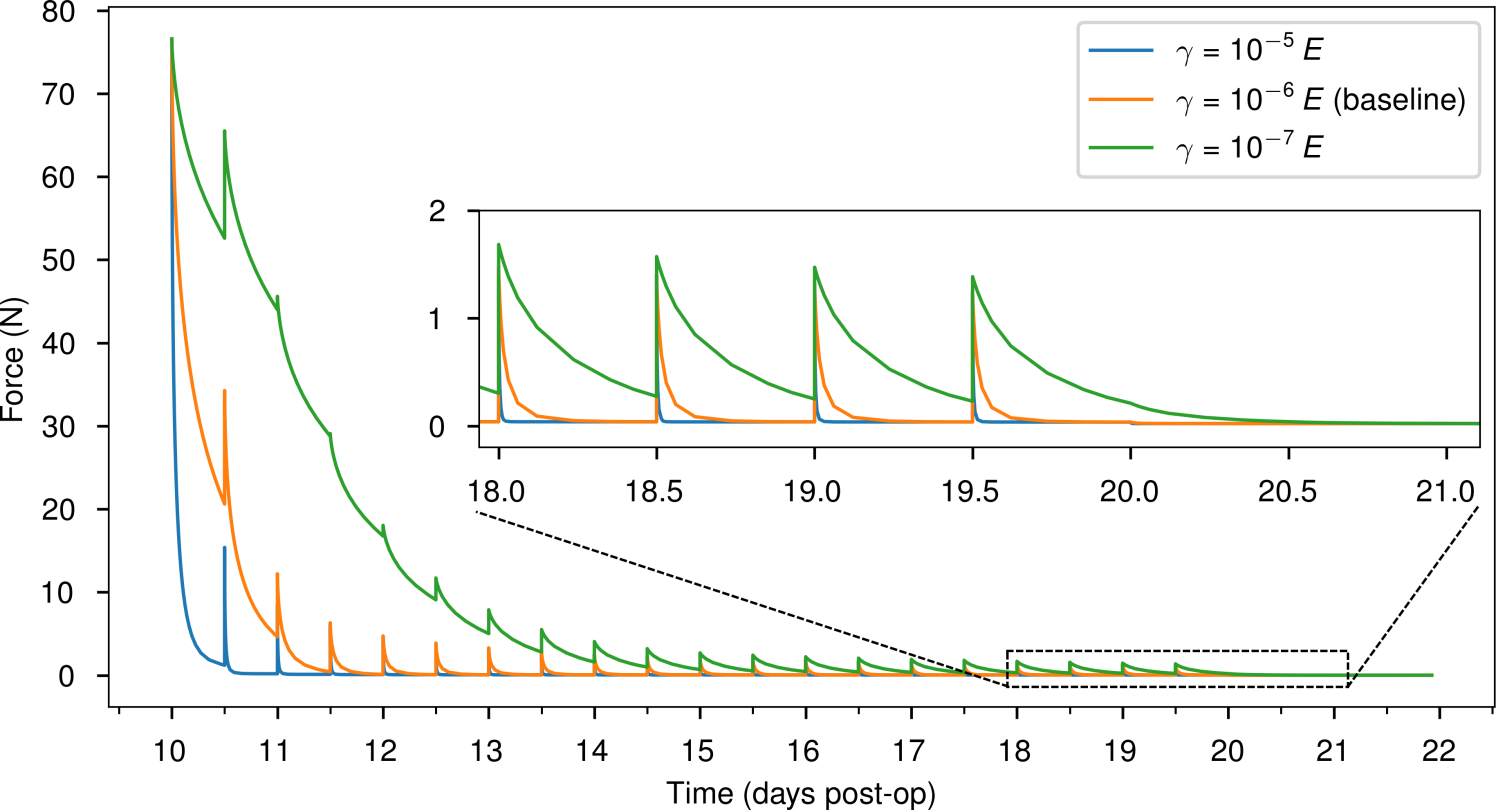
Evolution of the total reaction force (along the negative *y*-direction) acting on the top nodes on which the distraction displacement is applied.

The reaction forces acting on the simulated slice of the distraction plate display the desired viscoelastic relaxation behavior, corresponding roughly to forces measured for callus distraction [43] (Fig 13). As expected, lower viscosity (higher fluidity) leads to quicker force relaxation and vice versa. Over the course of the first four to five days of distraction, peak forces quickly diminish and become almost identical for all three cases of viscosity. This is expected behavior, as the strain induced in the soft tissue by each distraction increment decreases from step to step: The first distraction step stretches the soft tissue layer to 6.4-fold its original height from 0.05 mm to 0.32 mm (0.27 mm per step). In the next step, its height is increased to 0.59 mm, a mere 1.8-fold increase, then 0.86 mm (1.5-fold increase) etc.

#### Paracrine signaling

**Fig 14:**
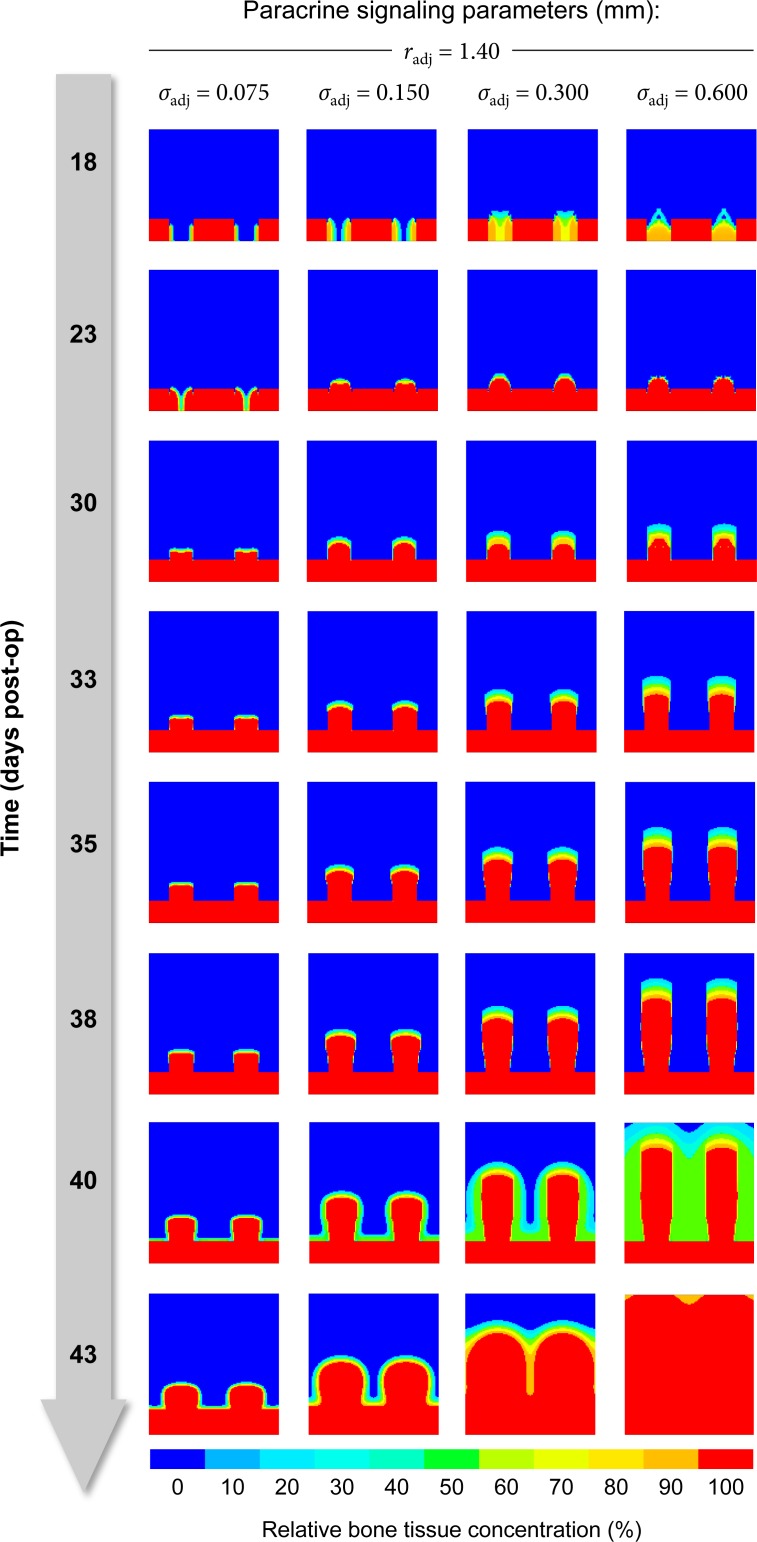
Paracrine signaling parameters control the propagation velocity of the bone front.

The parameters *σ*_adj_ and *r*_adj_ control how adjacent concentrations of bone and vascularity translate to the non-local biological stimuli *s*_b_ and *s*_v_. Larger values of *σ*_adj_, the standard deviation of the Gaussian convolution kernel *G*_adj_, increase the influence of distant tissue on the local differentiation process and can be interpreted as boosting diffusivity for signaling molecules. The value of *σ*_adj_ has a profound influence on the growth speed of both bone and vascularity, because it defines in which distance to existing bone and vasculature osteogenesis and angiogenesis can occur at which rate (Fig 14). Assuming the other parameters to be fixed, a value of 0.30 mm yields results that by and large resemble the *in vivo* observations.

The parameter *r*_adj_ specifies the support of the convolution kernel *G*_adj_. Tissue farther away than *r*_adj_ has no influence on local processes (i. e. *G*_adj_(***x***) ≡ 0 for ‖***x***‖ > *r*_adj_). While not strictly necessary, this optimization helps to reduce the computational and memory cost significantly, in particular in the 3D case and at high spatial resolutions. While experimenting with different combinations of *σ*_adj_ and *r*_adj_, we found a support of $r_{\mathrm{adj}}=\frac{7}{3}\sigma_{\mathrm{adj}}$ to be a viable trade-off between cost and possible drawbacks, e. g. artificially limiting the predicted growth speed.

#### Stimuli delay and memory

**Fig 15:**
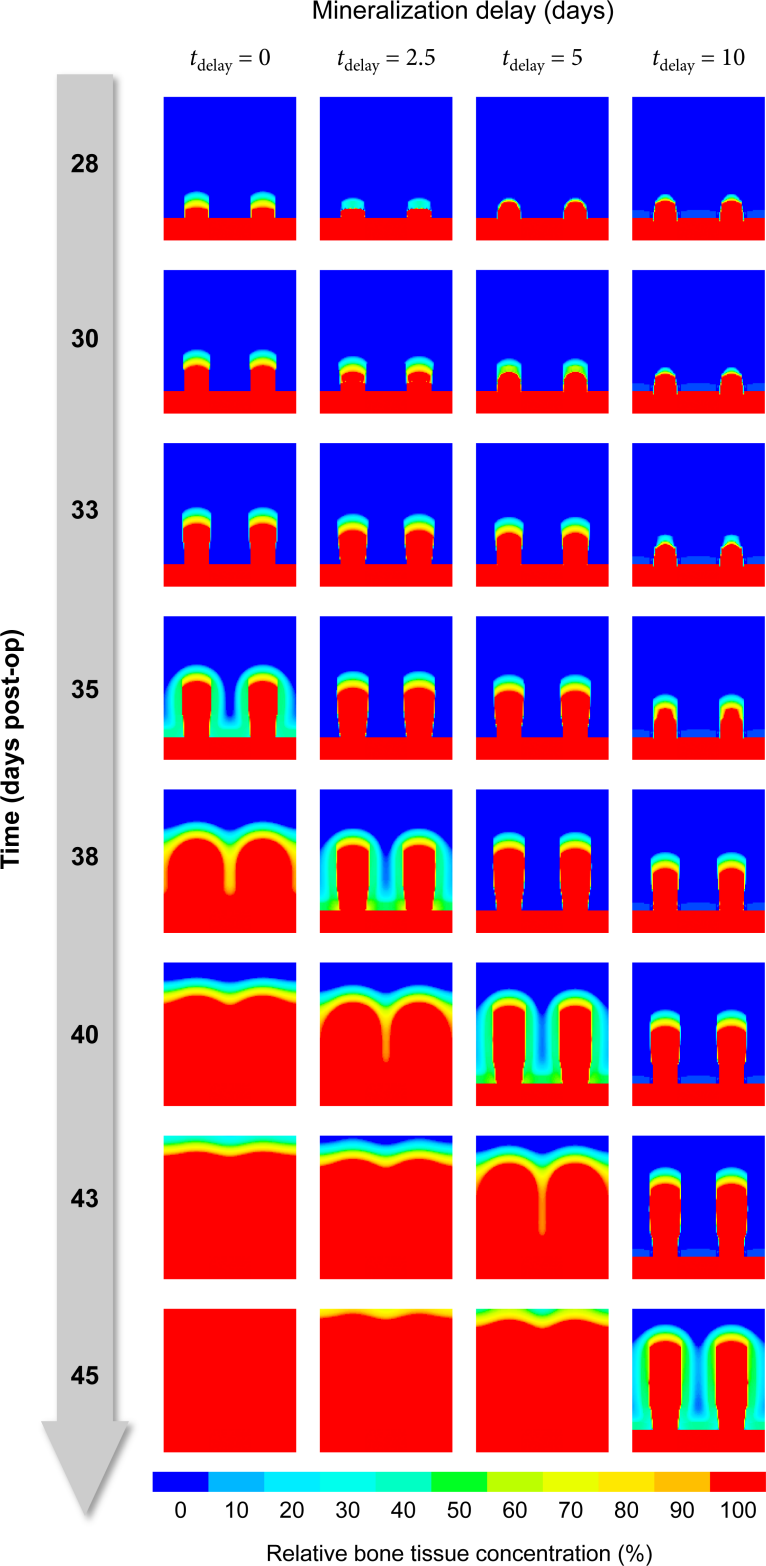
The parameter *t*_delay_ shifts the prediction temporally.

The parameter *t*_delay_ captures multiple processes responsible for the time-delay between stimulation and the occurrence of mineralized bone tissue. We know that its value should be on the order of days due to the delay caused by mineralization alone [55]. Simulations with *t*_delay_ = 0,2.5,5,10 days expectably predict almost identical ossification patterns, albeit shifted temporally corresponding to the value of *t*_delay_ (Fig 15).

**Fig 16:**
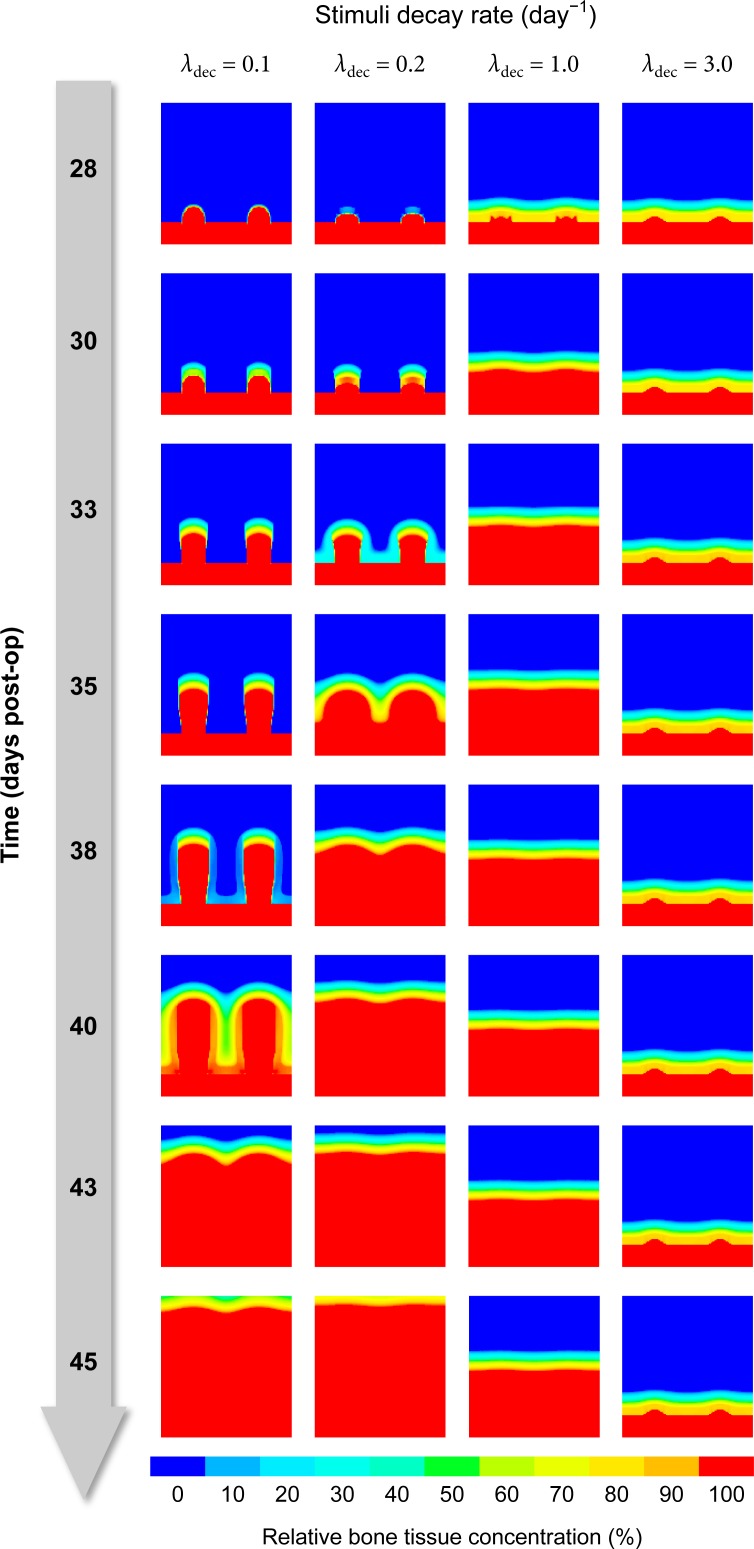
The stimuli decay rate needs to be low enough to allow the formation of bony cones.

Aside from this delay, there is also the (more obvious) effect of memorized stimulation, leading to sustained osteogenesis weeks after the last distraction step [32]. In the model, the parameter *λ*_decay_ describes how quickly the influence of past stimuli decreases. Fig 16 demonstrates that the decay rate must be low enough (≈ 0.1/day) to allow for the formation of pronounced bony cones above the drill holes as observed in the experiment and for bone formation to continue for the expected amount of time.

The third parameter controlling the influence of the strain history *t*_mem_ is the temporal analog to *r*_adj_ in that it limits the support of the exponential convolution kernel: Stimuli older than *t*_mem_ have no influence on current processes. For our choice of *λ*_decay_ = 0.1/day a value of *t*_mem_ = 45 days has proven to be sufficient to avoid significant truncation.

#### Distraction protocol

**Fig 17:**
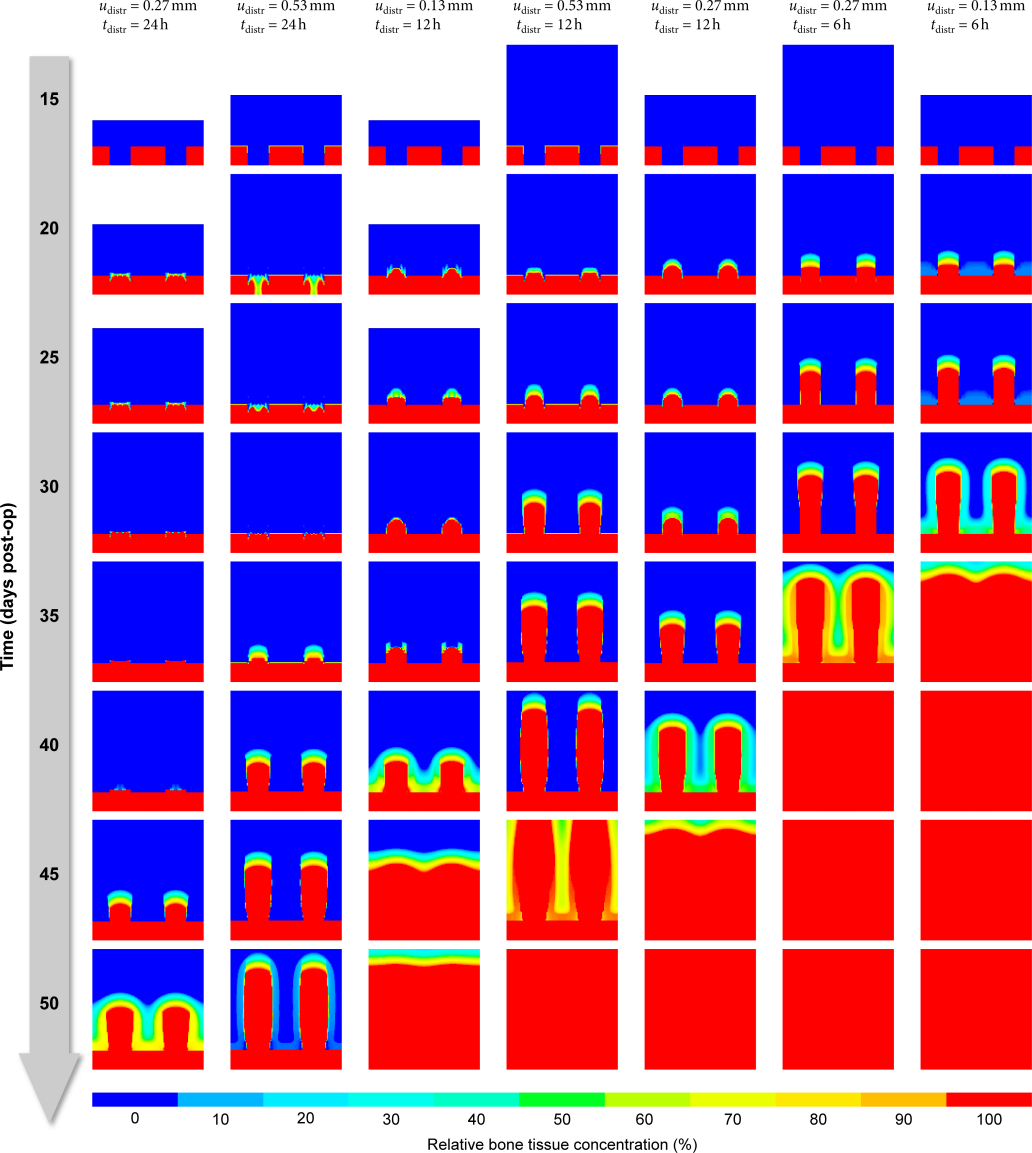
Comparison of seven different distraction protocols.

Varying the distraction increment *u*_distr_ as well as the distraction interval *t*_distr_ allows us to explore the impact of choosing between different distraction protocols on bone formation. Fig 17 compares seven different distraction protocols = combinations of *u*_distr_ and *t*_distr_), sorted left to right by “success,” i. e. how quickly the healing area is filled with bone. While there seems to be no single determining factor that predicts the rate of bone formation, a combination of the distraction rate *u*_distr_/*t*_distr_ and the “smoothness” of the protocol obviously correlates with the ossification pattern and speed: More frequent smaller distraction increments–and therefore smaller peak strains–produce significantly more bone considerably faster than other protocols.

## Discussion & conclusions

In this study, we introduced a novel computational model of lateral distraction osteogenesis based on the tissue differentiation hypothesis of Claes & Heigele and its further refinement and numerical implementation in the form of Simon et al.’s fracture healing model [33,34,38]. Aside from obvious changes, including a different geometry and adapted load and boundary conditions, we had to substantially extend the existing bone healing model in order to be able to describe the distraction procedure and the effects observed *in vivo*:

To describe stress relaxation and plastic deformation, instead of assuming linear quasi-static mechanics the new model features viscoplastic materials, requiring expensive transient and non-linear FE analyses. Consequently, tissue differentiation now depends on strain history instead of immediate stimulation alone, which also enables simulating effects such as calcification delay and continuing osteogenesis without mechanical stimulation thanks to the residual effects of “memorized” stimuli.

While Simon et al.’s model does consider appositional growth of bone and blood vessels, its implementation is tightly coupled to the discretized representation of the concentration fields, making predicted osteogenesis and angiogenesis rates depend on the chosen spatial mesh resolution. The convolution-based mesh-independent reformulation of non-local biological stimuli (“paracrine signaling”) we introduced in this study does not suffer from this issue and provides constant growth rates on sufficiently fine meshes [37]. To cope with the large plastic deformations and consequent mesh deformation, in particular during the first few distraction steps, we employ a remeshing and solution mapping scheme that recreates the FE mesh after each distraction step, ensuring a constant discretization quality. We then map both the biological (tissue concentrations) and the mechanical state (remaining elastic strains) to the new mesh.

After careful tuning of the newly introduced parameters, this enhanced model is indeed able to reproduce the main characteristics of the experimental results, indicating that the underlying tissue differentiation rules apply to distraction osteogenesis as well. The model predicts realistic average bone apposition rates of ≈ 100 μm/day. Just as *in vivo*, bony cones form above the drill holes, most likely due to enhanced vascularization as well as lower strains in these particular regions. The simulations further suggest that the vast amount of calcified bone tissue only appears after the distraction phase, which also qualitatively agrees with X-ray evidence from 4 weeks post-op (cf. Fig 1).

According to the model, higher distraction rates should be preferable to lower ones. Furthermore, frequent small increments generate more bone more quickly than strongly discontinuous, stepped distraction protocols. Such “smoother” protocols reduce peak strains (and stresses), keeping the effective stimuli in a more favorable moderate region, particularly during the initial distraction steps. A number of both *in vivo* and *in silico* studies confirm these predictions: Ilizarov suggested that the optimal distraction rate must be high enough to avoid premature union, but also low enough to avoid soft tissue damage [7]. The latter cannot be reproduced by our model, as it does not consider effects such as rupturing of collagen fibers in soft tissues or subjective factors like pain that limit the practical distraction rate. Illizarov, however, also agreed that small high-frequency steps were far superior to large increments. Aronson agreed that a more continuous distraction was preferable, but regarded two distractions per day as sufficient [56].

Experimenting with rabbits, Li et al. also arrived at the conclusion that increasing the distraction rate from 0.3 to 0.7 mm/day enhanced osteogenesis and revascularization, while further increasing it to 1.3 mm/day did not improve the result [57–59]. *In vivo* studies by Aarnes et al. and Mizuta et al. as well as the *in silico* study by Isakson et al. provide further support for the claim that smooth distraction may boost bone formation [21,60,61].

In the animal experiment, bone growth was also observed in the drill holes of the control group. Our model requires mechanical stimulation for osteogenesis and thus cannot replicate results where osteogenesis may have been triggered by trauma and/or inflammation effects alone. For the same reason we had to disable the simulation of bone resorption, as otherwise the cortical layer would have been partially resorbed during the latency period with no mechanical stimulation. Because we only care about a relatively short time period, ignoring remodeling should not, however, result in vastly different results.

The numerical model relies on a vast amount of, often highly uncertain, parameter values and the model extensions only introduce additional parameters. For calibrating the parameters, it does not help that the available experimental data is rather sparse (fluorescence labeling of calcified tissue at days 20 and 30 and post-mortem analyses), mostly qualitative and afflicted with a high degree of inter-individual variability. In light of the scarce data, we therefore had to rely on a mixture of educated guessing and systematic parameter variation to identify plausible values or at least guarantee that unknown parameters do not strongly affect the predicted growth patterns.

One serious drawback of OFAT (one-factor-at-a-time) sensitivity analysis is that it cannot discover interactions between parameters. We have shown, for instance, that the model is largely insensitive to the unknown viscoplastic material properties. This makes sense, insofar as tissue differentiation depends only on peak strain values, which are only affected slightly by the exact viscoplastic relaxation behavior. With a different sampling strategy (e. g. simply considering the current strain as the characteristic stimulus) the model might be much more sensitive to those unknown material properties.

As the parameters describing the non-local biological stimuli are unknown as well and probably cannot be measured even in principle, we adjusted the parameters to approximate the behavior (growth speed) of Simon et al.’s bone healing model, while at the same time achieving growth rates sufficient for the lateral distraction case.

Our implementation of the stimuli memory is purely phenomenological and offers no explanation of the underlying mechanisms, although a transcription-based memory might be able explain the observed effects [62–64]. The assumed exponential decay is pure speculation that fits the limited experimental data, but that might nevertheless turn out to be completely wrong on closer inspection backed by more precise data.

We assume furthermore that the mechanical properties of pure soft (connective) tissue stay constant. Preliminary experimental results of an *in vivo* study currently conducted at our institute suggests, however, that the visco-elastic behavior of the connective tissue changes over the course of the distraction phase, which would certainly influence the reaction forces and might also affect the mechanical stimuli. Future iteration of the model will need to take this new information, once available, into account.

Our solution method of decoupling the mechanical boundary-value problem from the biological initial-value problem using the method-of-lines seems to be justified as long as the simulated biological processes are “slow” compared to the mechanics, i. e. tissue differentiation within one distraction step does not significantly alter the material properties in the healing domain. While we have experimented with more sophisticated time-integration schemes, neither adaptive time-stepping nor implicit integrators seem to offer noteworthy advantages or turn out to be even more expensive than straightforward explicit fixed-step integration schemes. Simple predictor-corrector schemes like Heun’s method or lower-order Runge-Kutta methods however have the potential to allow approximately twice the time step size at comparable accuracy and cost per step and might therefore be viable options for future implementations.

Overall, we have achieved our goal of developing a numerical model that replicates the most prominent features of lateral distraction osteogenesis and that offers the possibility of performing *in silico* experiments to determine the effect of different distraction protocols. With that, we have also shown that the underlying tissue differentiation hypothesis, originally developed for fracture healing scenarios, is capable to describe distraction osteogenesis as well. In order to move further towards the overarching goal of creating a tool that can help both in basic research and, one day, in clinical practice, the next steps must focus on creating more reliable, quantitative data that can be used to calibrate and later validate our model more rigorously. Only then can we tackle the problem of transferring the model to more complicated real-world cases in order to optimize clinical procedures.

## Supporting information

S1 File Differentiation rules. Fuzzy logic membership functions and linguistic rules controlling tissue differentiation.
(PDF)

S2 File Mathematical notation. (PDF)
