# Supplementary material for: Simulating lateral distraction osteogenesis
Source: PLoS One. 2018 Mar 15;13(3):e0194500. doi: 10.1371/journal.pone.0194500 (PMC5854389; doi:10.1371/journal.pone.0194500)
Supplement: S1 File — Fuzzy logic membership functions and linguistic rules controlling tissue differentiation. (PDF) [file pone.0194500.s001.pdf]

S1 Differentiation rules

For approximating the tissue concentration rates of change we use the stand-alone fuzzy logic controller provided by MATLAB's Fuzzy Logic Toolbox (The MathWorks, Inc., Natick, MA), compiled as a shared C library with the following configuration:

Option	Value
Input variables	$c_\mathrm{w}, c_\mathrm{l}, c_\mathrm{c}, c_\mathrm{s}, c_\mathrm{v}, s_\mathrm{b}, s_\mathrm{v}, \gamma_\mathrm{peak}, \varepsilon_\mathrm{peak}, \gamma_\mathrm{eff}, \varepsilon_\mathrm{eff}$
Output variables	$\Delta c_\mathrm{w}, \Delta c_\mathrm{c}, \Delta c_\mathrm{v}$
Logical AND operator	$\min$
Logical OR operator	$\max$
Logical implication	$\min$ (Mamdani implication)
Logical aggregation	$\max$
Defuzzification	Center of area

Linguistic rules

The fuzzy logic controller evaluates the following 27 linguistic rules to determine the values of $\Delta c_\mathrm{w}, \Delta c_\mathrm{c}$ and Δc_v:

Rule no.	If ...	then ...
1	$\varepsilon_\mathrm{peak}$ is negative destructive	Δc_w is negative and<br>Δc_c is negative and<br>Δc_v is negative
2	$\varepsilon_\mathrm{peak}$ is positive destructive	Δc_w is negative and<br>Δc_c is negative and<br>Δc_v is negative
3	γ_peak is very high	Δc_w is negative and<br>Δc_c is negative and<br>Δc_v is negative
4	$\varepsilon_\mathrm{peak}$ is not negative destructive	Δc_w is about zero and<br>Δc_c is about zero and<br>Δc_v is about zero
5	$\varepsilon_\mathrm{peak}$ is not positive destructive	Δc_w is about zero and<br>Δc_c is about zero and<br>Δc_v is about zero
6	c_v is low and<br>s_v is not low and<br>$\varepsilon_\mathrm{peak}$ is not negative high and<br>γ_peak is not high	Δc_v is positive

7	c_{v} is medium and<br>s_{v} is not low and<br>$\varepsilon_{\mathrm{peak}}$ is not negative high and<br>γ_{peak} is not high	Δc_{v} is positive
8	c_{v} is high and<br>$\varepsilon_{\mathrm{peak}}$ is not negative high and<br>γ_{peak} is not high	Δc_{v} is positive
9	c_{c} is low and<br>c_{v} is high and<br>s_{v} is high and<br>$\varepsilon_{\mathrm{eff}}$ is negative medium<br>γ_{eff} is medium	Δc_{w} is positive
10	c_{c} is low and<br>c_{v} is high and<br>s_{v} is high and<br>$\varepsilon_{\mathrm{eff}}$ is positive medium<br>γ_{eff} is medium	Δc_{w} is positive
11	c_{b} is not high and<br>c_{c} is low and<br>$\varepsilon_{\mathrm{peak}}$ is negative high and<br>γ_{peak} is not very high	Δc_{c} is positive
12	c_{b} is not high and<br>c_{c} is low and<br>$\varepsilon_{\mathrm{peak}}$ is negative medium and<br>γ_{peak} is not very high	Δc_{c} is positive
13	c_{c} is not low and<br>$\varepsilon_{\mathrm{peak}}$ is negative high and<br>γ_{peak} is not very high	Δc_{c} is positive
14	c_{c} is not low and<br>$\varepsilon_{\mathrm{peak}}$ is negative medium and<br>γ_{peak} is not very high	Δc_{c} is positive
15	c_{c} is not low and<br>c_{v} is not low and<br>s_{b} is not low and<br>$\varepsilon_{\mathrm{eff}}$ is negative medium and<br>γ_{eff} is low	Δc_{w} is positive
16	c_{c} is not low and<br>c_{v} is not low and<br>s_{b} is not low and	Δc_{c} is negative

	ε_{peak} is negative medium and<br>γ_{peak} is low	
17	c_c is not low and<br>c_v is not low and<br>s_b is not low and<br>ε_{eff} is negative high and<br>γ_{eff} is low	Δc_w is positive
18	c_c is not low and<br>c_v is not low and<br>s_b is not low and<br>ε_{peak} is negative high and<br>γ_{peak} is low	Δc_c is negative
19	c_c is not low and<br>c_v is not low and<br>s_b is not low and<br>ε_{eff} is negative high and<br>γ_{eff} is medium	Δc_w is positive
20	c_c is not low and<br>c_v is not low and<br>s_b is not low and<br>ε_{peak} is negative high and<br>γ_{peak} is medium	Δc_c is negative
21	c_c is not low and<br>c_v is not low and<br>s_b is not low and<br>ε_{eff} is negative medium and<br>γ_{eff} is medium	Δc_w is positive
22	c_c is not low and<br>c_v is not low and<br>s_b is not low and<br>ε_{peak} is negative medium and<br>γ_{peak} is medium	Δc_c is negative
23	c_c is low and<br>c_v is not low and<br>c_b is high and<br>s_b is high and<br>ε_{eff} is negative medium and<br>γ_{eff} is medium	Δc_w is positive
24	c_c is low and	Δc_c is negative

	c_v is not low and<br>c_b is high and<br>s_b is high and<br>$\varepsilon_\mathrm{peak}$ is negative medium and<br>γ_peak is medium	
25	c_c is low and<br>c_v is not low and<br>c_b is high and<br>s_b is high and<br>ε_eff is negative medium and<br>γ_eff is low	Δc_w is positive
26	c_c is low and<br>c_v is not low and<br>c_b is high and<br>s_b is high and<br>$\varepsilon_\mathrm{peak}$ is negative medium and<br>γ_peak is low	Δc_c is negative
27	ε_eff is about zero and<br>γ_eff is very low	Δc_w is negative

Note that Rule 27 (bone resorption due to understimulation) is evaluated separately from the other rules as a post-processing step in order to determine resorption rates for both woven and lamellar bone (for implementation details, please refer to the main text as well as Frank Niemeyer's PhD thesis [pages 329 and 330; DOI: 10.18725/OPARU-2961]).

Membership functions

The fuzzy sets the linguistic rules refer to are defined as the following trapezoidal membership functions:

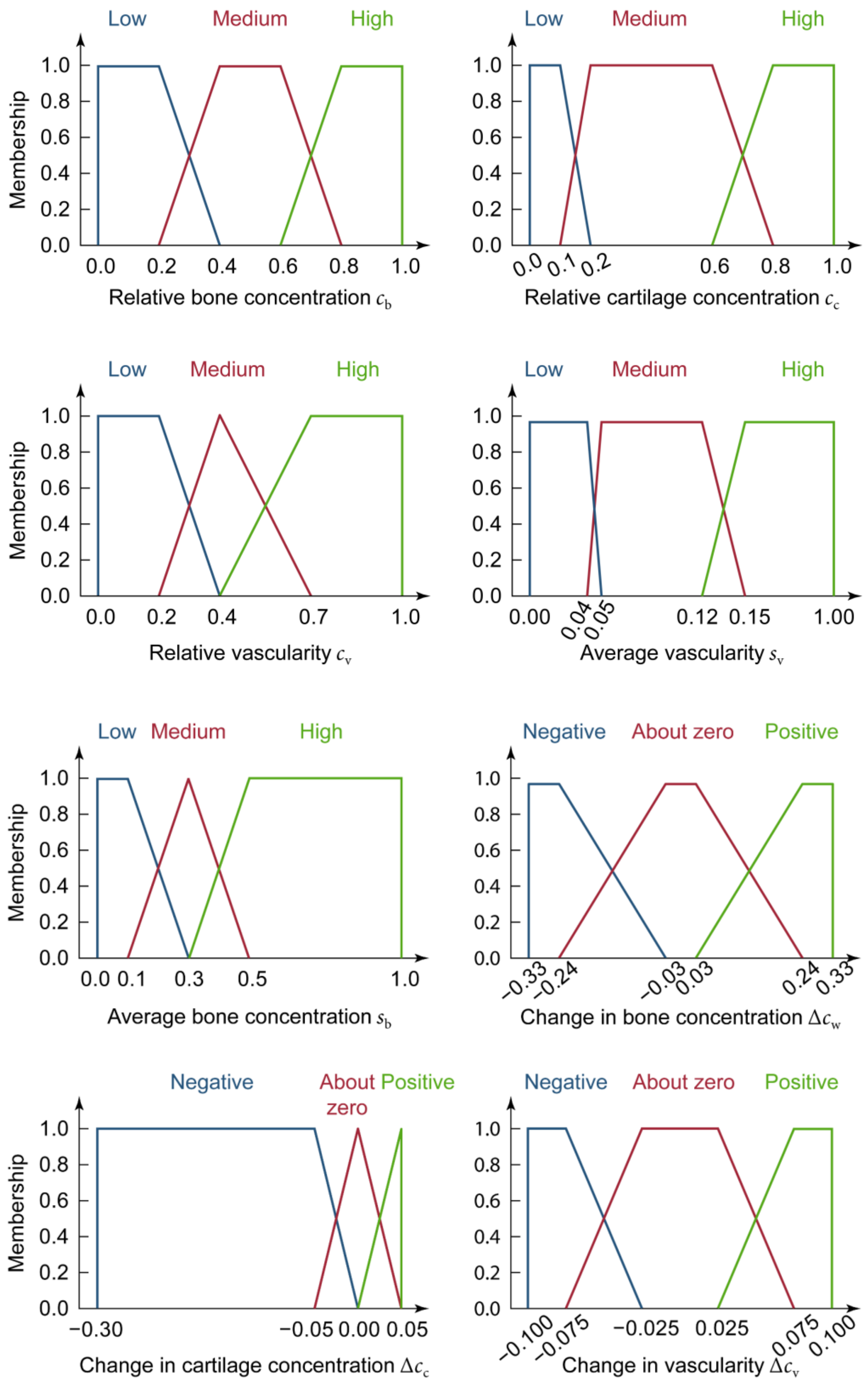
Low
Medium
High
Membership
Relative bone concentration c_b
Low
Medium
High
Relative cartilage concentration c_c
Low
Medium
High
Membership
Relative vascularity c_v
Low
Medium
High
Average vascularity s_v
Low
Medium
High
Membership
Average bone concentration s_b
Negative
About zero
Positive
Change in bone concentration Δc_w
Negative
About zero
Positive
Membership
Change in cartilage concentration Δc_c
Negative
About zero
Positive
Change in vascularity Δc_v

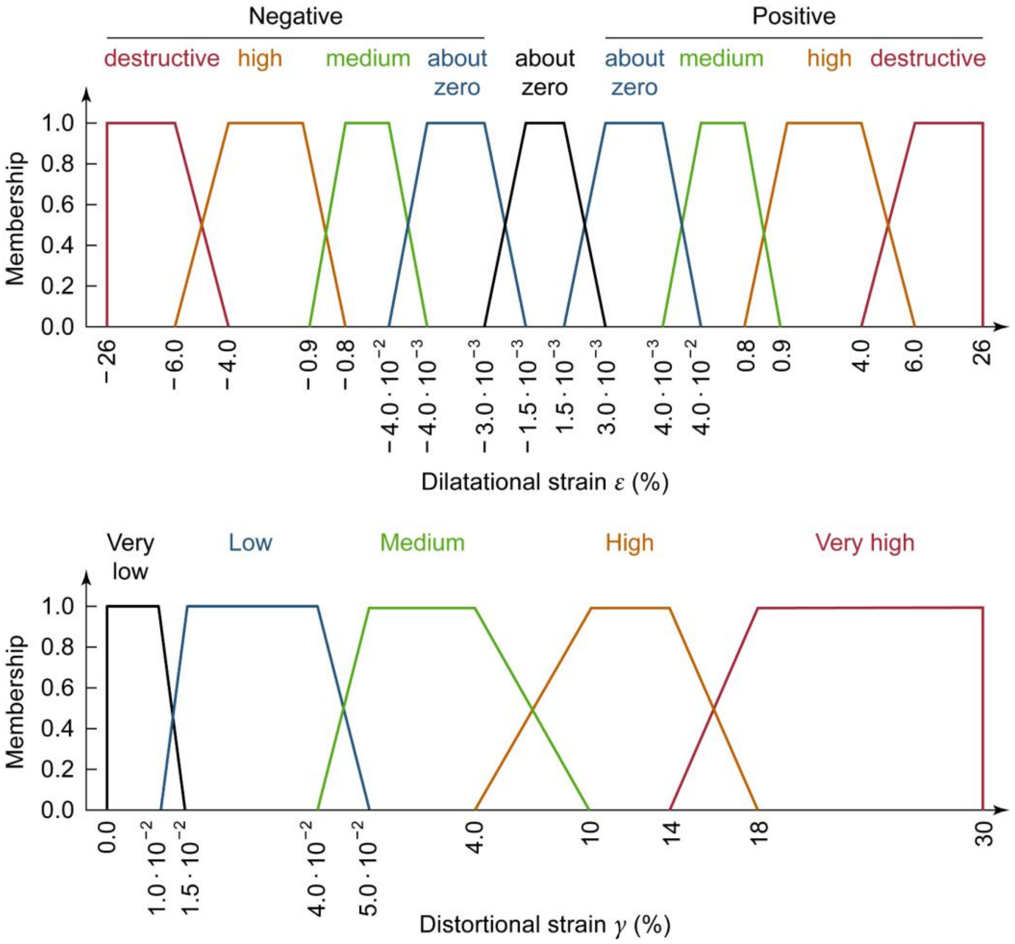

Negative
Positive
destructive
high
medium
about zero
about zero
about zero
medium
high
destructive
Membership
1.0
0.8
0.6
0.4
0.2
0.0
− 26
− 6.0
− 4.0
− 0.9
− 0.8
− 4.0 · 10^−2
− 4.0 · 10^−3
− 3.0 · 10^−3
− 1.5 · 10^−3
1.5 · 10^−3
3.0 · 10^−3
4.0 · 10^−3
4.0 · 10^−2
0.8
0.9
4.0
6.0
26
Dilatational strain ε (%)
Very low
Low
Medium
High
Very high
Membership
0.0
1.0 · 10^−2
1.5 · 10^−2
4.0 · 10^−2
5.0 · 10^−2
4.0
10
14
18
30
Distortional strain γ (%)
