# Supplementary material for: Simulating lateral distraction osteogenesis
Source: PLoS One. 2018 Mar 15;13(3):e0194500. doi: 10.1371/journal.pone.0194500 (PMC5854389; doi:10.1371/journal.pone.0194500)
Supplement: S2 File — (PDF) [file pone.0194500.s002.pdf]

S2 Mathematical notation

Anonymous functions, function literals, “lambda expressions”

An expression of the form

$$(x_1, x_2, \dots, x_n) \mapsto f(x_1, x_2, \dots, x_n)$$

defines an anonymous n-ary function. The function’s type (“domain” and “codomain”) are inferred from the context.

Partial application

Given an n-ary function

$$f: (U_1, U_2, \dots, U_n) \to V$$

the expression

$$f(x_1, \cdot, \cdot, \dots, \cdot) = g$$

fixes the first parameter of f, mapping f to a (n-1)-ary function

$$g: (U_2, U_3, \dots, U_n) \to V$$
$$(x_2, x_3, \dots, x_n) \mapsto f(x_1, x_2, \dots, x_n)$$

Example: Let

$$f(x, y) = \sin(x)\cos(y)$$

Then

$$g = f(2\pi, \cdot) = x \mapsto \sin(2\pi)\cos(x).$$
